# Adiponectin suppresses amyloid-β oligomer (AβO)-induced inflammatory response of microglia via AdipoR1-AMPK-NF-κB signaling pathway

**DOI:** 10.1186/s12974-019-1492-6

**Published:** 2019-05-25

**Authors:** Min Jian, Jason Shing-Cheong Kwan, Myriam Bunting, Roy Chun-Laam Ng, Koon Ho Chan

**Affiliations:** 1 Department of Medicine, LKS Faculty of Medicine, The University of Hong Kong, 8/F, 21 Sassoon Road, Pokfulam, Hong Kong, Special Administrative Region of China; 2 Neuroimmunology and Neuroinflammation Research Laboratory, LKS Faculty of Medicine, The University of Hong Kong, Pokfulam, Hong Kong, Special Administrative Region of China; 3 Research Center of Heart, Brain, Hormone and Healthy Aging, LKS Faculty of Medicine, The University of Hong Kong, Pokfulam, Hong Kong, Special Administrative Region of China; 4 Hong Kong University Alzheimer’s Disease Research Network, LKS Faculty of Medicine, The University of Hong Kong, Pokfulam, Hong Kong, Special Administrative Region of China; 5 Department of Medicine, The University of Hong Kong, 4/F, Professorial Block, Queen Mary Hospital, 102 Pokfulam Road, Pokfulam, Hong Kong, Special Administrative Region of China

**Keywords:** Alzheimer’s disease, Adiponectin, Microglia, Neuroinflammation

## Abstract

**Background:**

Microglia-mediated neuroinflammation is important in Alzheimer’s disease (AD) pathogenesis. Extracellular deposition of β-amyloid (Aβ), a major pathological hallmark of AD, can induce microglia activation. Adiponectin (APN), an adipocyte-derived adipokine, exerts anti-inflammatory effects in the periphery and brain. Chronic APN deficiency leads to cognitive impairment and AD-like pathologies in aged mice. Here, we aim to study the role of APN in regulating microglia-mediated neuroinflammation in AD.

**Methods:**

Inflammatory response of cultured microglia (BV2 cells) to AβO and effects of APN were studied by measuring levels of proinflammatory cytokines (tumor necrosis factor α [TNFα] and interleukin-1β [IL-1β]) in cultured medium before and after exposure to AβO, with and without APN pretreatment. Adiponectin receptor 1 (AdipoR1) and receptor 2 (AdipoR2) were targeted by small interference RNA. To study the neuroprotective effect of APN, cultured HT-22 hippocampal cells were treated with conditioned medium of AβO-exposed BV2 cells or were co-cultured with BV2 cells in transwells. The cytotoxicity of HT-22 hippocampal cells was assessed by MTT reduction. We generated APN-deficient AD mice (APN^−/−^5xFAD) by crossing APN-knockout mice with 5xFAD mice to determine the effects of APN deficiency on microglia-mediated neuroinflammation in AD.

**Results:**

AdipoR1 and AdipoR2 were expressed in BV2 cells and microglia of mice. Pretreatment with APN for 2 h suppressed TNFα and IL-1β release induced by AβO in BV2 cells. Additionally, APN rescued the decrease of AMPK phosphorylation and suppressed nuclear translocation of nuclear factor kappa B (NF-κB) induced by AβO. Compound C, an inhibitor of AMPK, abolished these effects of APN. Knockdown of AdipoR1, but not AdipoR2 in BV2 cells, inhibited the ability of APN to suppress proinflammatory cytokine release induced by AβO. Moreover, pretreatment with APN inhibited the cytotoxicity of HT-22 cells co-cultured with AβO-exposed BV2 cells. Lastly, APN deficiency exacerbated microglia activation in 9-month-old APN^−/−^5xFAD mice associated with upregulation of TNFα and IL-1β in the cortex and hippocampus.

**Conclusions:**

Our findings demonstrate that APN inhibits inflammatory response of microglia to AβO via AdipoR1-AMPK-NF-κB signaling, and APN deficiency aggravates microglia activation and neuroinflammation in AD mice. APN may be a novel therapeutic agent for inhibiting neuroinflammation in AD.

**Electronic supplementary material:**

The online version of this article (10.1186/s12974-019-1492-6) contains supplementary material, which is available to authorized users.

## Background

Alzheimer’s disease (AD), the most common cause of dementia in the elderly, is clinically characterized by progressive cognitive impairment typically memory decline [1]. While the pathophysiology for AD remains uncertain, intracellular neurofibrillary tangles and extracellular deposition of amyloid plaques are two major histopathological hallmarks of AD [2]. β-Amyloid (Aβ) is a short peptide generated from sequential proteolytic cleavage of amyloid precursor protein (APP) and forms Aβ monomers, soluble Aβ oligomer (AβO), and insoluble fibrillar Aβ [3, 4]. Accumulating evidence suggests that soluble AβO may be the most neurotoxic forms underlying AD [5, 6]. AβO can directly cause neuronal damage, synaptic injury, and memory loss [7–9]; induce tau hyperphosphorylation; and initiate the neuroinflammation [10]. Therefore, targeting soluble AβO might be an optimal immunotherapeutics for AD. Increasing evidence suggests that neuroinflammation mediated by glial cells, including both microglia and astrocytes, contributes to AD pathogenesis [11]. Whole-genome analyses support that microglia-specific triggering receptor expressed on myeloid cells 2 (TREM2)-mediated inflammatory response is associated with AD [12]. An innate immune response can be triggered by misfolded and aggregated proteins binding to pattern recognition receptors on microglia and astrocytes to induce the release of inflammatory cytokines, contributing to AD progression and severity [13].

Microglia, the resident phagocyte of the central nervous system (CNS), is pivotal for surveillance in the CNS. They respond rapidly to pathological triggers, such as neuronal death or protein aggregates, and remove them via phagocytosis and degradation [14]. It is widely believed that acute microglia activation is beneficial in neuroinflammatory conditions by promoting clearance of neurotoxic agents and restoration of tissue homoeostasis [15, 16]. However, upon chronic activation, microglia became dysfunctional characterized by morphology changes, upregulation of inflammatory cytokines and chemokines, resulting in CNS destabilization and neuronal degeneration [17, 18]. Emerging studies have demonstrated that microglia-mediated neuroinflammation plays an important role in the pathogenesis of AD. These cells appear to react to amyloid plaques and are involved in clearance of Aβ [13, 15]. A variety of inflammatory mediators are linked to the progression of AD. Tumor necrosis factor α (TNFα), interleukin-1β (IL-1β), and interferon-γ (IFN-γ) enhance Aβ production [19, 20]. In addition, TNFα treatment can reduce expression of CD36 on microglia and impair Aβ clearance [14]. Moreover, inhibition of IL-12 and IL-23 signaling from microglia in APP/PS1 mouse model decreases cerebral amyloid load [21]. Therefore, modulating microglia-mediated neuroinflammation can be a promising therapeutic target for AD.

Adiponectin (APN) is an adipokine secreted predominantly from adipocytes and circulates as oligomers, including full-length trimers, hexamers, high molecular weight (HMW) multimers, and globular adiponectin (gAPN) in the blood [22]. The biological effects of APN are mediated through two receptors, AdipoR1 and AdipoR2, which are highly expressed in the liver [23, 24], muscle [25], heart [26], and adipose tissue [27]. Numerous studies have shown that APN plays an important role in glucose and lipid metabolism [28], insulin sensitization [29], vascular protection [30] and exerting anti-atherosclerosis effects [31, 32] and anti-inflammatory effects [33–36] in the periphery. AdipoR1 and AdipoR2 are also expressed in the CNS at the cortex, the hippocampus, the hypothalamus, amygdala, and brain microvessels [22]. However, the function of APN in the CNS is still not fully understood. Only low molecular weight forms of APN, trimeric and hexameric APN, can cross the blood–brain barrier and exert their effects in the CNS [22, 37]. It has been shown that APN mediates the beneficial effects of physical exercise [38] and enriched environment [39] on depression. APN also facilitates contextual fear extinction through AdipoR2 in the dentate gyrus (DG) [40] and regulates anxiety-related behavior through AdipoR1 in ventral tegmental area (VTA) [41]. APN-deficient mice have more severe CNS inflammation and demyelination when induced to develop experimental autoimmune encephalomyelitis (EAE), an animal model of human multiple sclerosis, compared to wild-type mice [42, 43]. It has also been reported that APN can decrease neuronal apoptosis and oxidative stress [44] and improve neurobehavioral function in mice subjected to cerebral ischemia [45]. A recent study showed that ICV injection of gAPN exerts direct anti-inflammatory effects on microglia by reducing proinflammatory cytokine synthesis in vivo [46]. Our previous study also suggests that APN has beneficial effects in AD. APN is protective against cytotoxicity of human neuroblastoma cells (SH-SY5Y) expressing mutant APP (Sw-APP) under oxidative stress through APPL1-mediated AMPK activation, associated with suppression of NF-κB activation [47]. We also showed that aged mice with chronic APN deficiency had cognitive impairment associated with AD-like pathologies and [48]. APN may be a novel therapy for AD. However, it is unclear whether APN can modulate microglia-mediated neuroinflammation in AD.

In the present study, we studied whether APN can modulate neuroinflammatory response of microglia in AD in vitro and in vivo models. We found that APN suppressed microglial neuroinflammatory response induced by AβO via AdipoR1-AMPK-NF-κB signaling pathway in BV2 cells. Importantly, APN inhibited the cytotoxicity of HT-22 hippocampal cells co-cultured with AβO-treated BV2 cells. Moreover, we showed that APN deficiency increased microglia activation associated with upregulation of TNFα and IL-1β in the cortex and hippocampus of 5xFAD mice. Taken together, our findings suggest that APN inhibits microglia-mediated neuroinflammation in AD.

## Methods

### Animal

5xFAD transgenic mice [49] were a gift from Dr. Durairajan S. S. Kumar. 5xFAD transgenic mice were crossed with C57BL/6 N mice to generate the heterozygous 5xFAD transgenic mice and wild-type littermate controls. APN^−/−^ mice were previously described [48]. 5xFAD transgenic mice were crossed to APN^−/−^ mice to generate APN^−/−^5xFAD mice. Genotyping was performed by polymerase chain reaction (PCR) of tail DNA as described previously [50]. All male mice (4–5 mice per cage) were maintained until 9 months old with standard conditions (23 ± 2 °C, 60–70% relative humidity, 12 h light/dark cycle,) provided with free access to food and water in the Laboratory Animal Unit of the University of Hong Kong. All animal studies were approved by the Committee on the Use of Live Animals in Teaching and Research of the University of Hong Kong.

### Cell culture and drug treatment

Murine BV2 microglia cells were a generous gift from Dr. Nicolai Savaskan (Universitätsklinikum Erlangen (UKER), Germany), and HT-22 hippocampal cells were obtained from Dr Kiren Rockenstein (Salk Institute, USA). BV2 cells and HT-22 cells were cultured in Dulbecco’s modified Eagle’s medium (DMEM) (Gibco) with 10% fetal bovine serum (FBS) (Invitrogen, USA) and 1% penicillin/streptomycin (Gibco). The cells were grown in a humidified incubator at 37 °C with 5% CO2. BV2 cells were pretreated with APN (10 μg/ml) (Antibody and Immunoassay Services, HKU) or Compound C (10 μM) (Calbiochem, USA) for 2 h and then treated with AβO (10 μM) for 24 h in serum-free culture medium. The cellular morphology of BV2 cells was observed by light microscopy with phase contrast (Olympus IX70, Olympus America, Inc.).

### Aβ oligomer (AβO) preparation

AβO were prepared as previously described [19]. In brief, 1 mg of Aβ_42_ peptide (GL Biochem, Shanghai) was dissolved in 221.7 μl cold HFIP (1,1,1,3,3,3-hexafluoro-2-propanol) (Sigma-Aldrich) to a concentration of 1 mM. The solution was incubated at room temperature (RT) for 1 h, and then placed on ice for 10 min. After incubation, the solution was aliquoted into non-siliconized microcentrifuge tubes (100 μl solution containing 0.45 mg Aβ_42_) and then dried overnight at RT. The residues were dissolved in 20 μl dimethyl sulfoxide (DMSO) and then added with F12 medium to obtain a 100 μM stock solution. The solution was incubated at 4 °C overnight and then centrifuged at 14,000×*g* for 10 min at 4 °C. Then, the Aβ oligomers were presented in the supernatant. The presence of Aβ oligomers was confirmed by immunoblot using anti-Aβ antibody (1:1000, BioLegend).

### AdipoR1 and AdipoR2 siRNA transfections

Mouse AdipoR1 and AdipoR2 siRNAs and non-targeting control siRNA were purchased from Santa Cruz Biotechnology. BV2 cells were seeded in a six-well tissue culture plate until 80% confluency. Then, BV2 cells were transfected with siRNA using lipofectamine 3000 reagent (Invitrogen, USA). The mixture of siRNA duplex and reagent was diluted in Opti-MEM medium (Gibco) and incubated at RT for 45 min. Then, the siRNA duplex and reagent mixture were added to BV2 cell. After 6-h incubation, medium containing siRNA was removed and cells were further cultured for 18 h before using in experiments and analysis.

### Cytokine ELISA

The concentrations of TNFα and IL-1β in culture medium were examined by Mouse Quantikine ELISA Kits according to the manufacturer’s protocol (R&D Systems). The optical density of each well at 450 nm was determined by a CLARIO star microplate reader (BMG LABTECH, Germany).

Production of TNFα and IL-1β were assessed in tissue homogenates. Briefly, frozen cortex and hippocampus were incubated in ice-cold lysis buffer (Cell Signaling Technology, USA) with PMSF for 30 min and then sonicated 3 × 15 s with a 2-min interval between each sonication in ice-cold lysis buffer. Samples were centrifuged at 14,000×*g* at 4 °C for 20 min to remove any insoluble materials, including nuclei and large debris, and the cytosolic protein concentration in supernatants was determined by Bradford test (BioRad, USA). Samples were then assessed in duplicate via RayBio® Mouse TNFα ELISA Kit (RayBiotech, Inc., USA) and IL-1β Mouse Quantikine ELISA Kits (R&D Systems) according to the manufacturer’s protocol. The concentration (pg/ml) of cytokines was normalized to total protein content (pg/mg of protein).

### Reverse transcriptase polymerase chain reaction for expression of adiponectin receptors

Total RNA was extracted from BV2 cells using Trizol reagent (Ambion, Invitrogen) with a DNase (Promega, Madison, WI, USA) treatment according to the manufacturer’s instructions. cDNA synthesis from 1 μg of total RNA in a reaction volume of 20 μl was performed with ImProm-IITM Reverse Transcription System (Promega, Madison, WI, USA) according to the manufacturer’s instructions. PCR amplification with specific primers was utilized following thermal cycling: 95 °C for 10 min, 35 cycles of denaturing at 95 °C for 1 min, annealing at 64 °C for 1 min, elongation at 72 °C for 1 min, final extension at 72 °C for 7 min and holding at 4 °C. PCR products were electrophoresed in 1% agarose gels. All samples were normalized to the housekeeping gene glyceraldehyde 3-phosphate dehydrogenase (GAPDH). The following primers were used: AdipoR1: forward (5′-AACTGGACTATTCAGGGATTGC-3′), reverse (5′-ACCATAGAAGTGGACGAAAGC-3′); AdipoR2: forward (5′-CCACCATAGGGCAGATAGG-3′), reverse (5′-TGAACAAAGGCACCAGCAA-3′); GAPDH: forward (5′-AAGCCCATCACCATCTTCCAG-3′), reverse (5′-AGAAGACTGTGGATGGCCCCT-3).

### Cytosolic and nuclear protein isolation

Cytosolic protein from BV2 cells was isolated as described previously [48]. Briefly, BV2 cells were washed with ice-cold phosphate-buffered saline (PBS) and lysed with 100 μl ice-cold lysis buffer (Cell Signaling Technology, USA) for 20 min with gentle shaking. Then, the lysates were centrifuged at 14,000×*g* for 10 min at 4 °C, followed by collecting supernatant within cytosolic protein fraction. The protein concentration was quantified using the Bradford assay (BioRad, USA).

Nuclear protein isolation protocol was performed with a nuclear extraction kit (Panomics, Inc.; Beijing, China) according to the manufacturer’s protocol as described earlier [47]. Briefly, BV2 cells were washed with ice-cold PBS and lysed in Buffer A working reagent containing DTT, protease inhibitor, and phosphate inhibitor for 10 min on ice. Each sample was transferred to a microcentrifuge tube and centrifuged at 14,000×*g* for 3 min at 4 °C. After removing the supernatant, Buffer B working reagent containing DTT, protease inhibitor, and phosphate inhibitor was added to the pellet and the microcentrifuge tubes were vortexed at the highest setting for 10 s. The pellet was detached from the microcentrifuge tube wall and incubated on ice for 1 h. The nuclear extraction was collected as supernatant. The protein concentration was quantified using the DC assay (BioRad, USA).

### Western blot analysis

Cell homogenates (20 μg/well) were loaded onto 10% SDS polyacrylamide gels in denaturing conditions at 80 mA for 90 min and transferred electrophoretically (100 mA/blot, 2 h; Power Pack; Bio-Rad Laboratories, Inc., USA) to polyvinylidene fluoride (PVDF) membrane. Immunoblotting was performed as described previously [48]. Nonspecific binding was blocked with 5% non-fat milk powder in Tris-buffered saline-Tween containing 0.1% Tween-20 (PBS-T) for 1 h. Primary antibodies including rabbit anti-AdipoR1 (1:1000, Abcam, Cambridge, MA, USA), rabbit anti-AdipoR2 (1:1000, Boster Biological Technology, USA), rabbit anti-AMPK (1:1000, Cell Signaling Tech. Inc., USA), rabbit anti-p-AMPK^T172^ (1:1000, Cell Signaling Tech. Inc., USA), rabbit anti-α-Tubluin (1:5000, Cell Signaling Tech. Inc., USA), rabbit anti-p-NF-κB p65^S536^ (1:1000, Cell Signaling Tech. Inc., USA), rabbit anti-NF-κB p65 (1:1000, Cell Signaling Tech. Inc., USA), rabbit anti-p-IκBα (Ser32) (1:1000, Cell Signaling Tech. Inc., USA), mouse anti-IκBα (1:1000, Cell Signaling Tech. Inc., USA) antibody were incubated at 4 °C overnight, followed by HRP-conjugated secondary antibodies (goat anti-rabbit, 1:5000 or rabbit anti-mouse, 1:5000; Dako, Glostrup, Denmark) at RT for 1 h. The immunoblot signals were visualized by Westernbright Quantum HRP substrate (advansta, USA).

### Immunocytochemistry

BV2 cells were seeded on glass chamber slides at a concentration of 1 × 10^4^ cells/well overnight, and then washed with phosphate-buffered saline (PBS) and fixed with 4% paraformaldehyde (Sigma-Aldrich) for 20 min at RT. The slides were rinsed with PBS and incubated with 0.05% Triton X-100 in PBS for 15 min at RT, followed with blocking solution (90% PBS, 10% goat serum, and 0.05% Triton X-100) at RT for 1 h. Slides were then incubated with mouse anti-Iba-1 (1:500, Abcam, Cambridge, MA, USA) and rabbit anti-AdipoR1 (1:500, Abcam, Cambridge, MA, USA) or goat anti-AdipoR2 (1:500, Abcam, Cambridge, MA, USA) at 4 °C overnight. After incubation, the cells were washed with 0.05% Triton X-100 in PBS three times and incubated with appropriate Alexa-Fluor-conjugated secondary antibody (Thermo Fisher Scientific, USA) at RT for 1 h. The coverslips were mounted with by slow fade® anti-fade DAPI reagent (Lifetech, US). The fluorescent images were captured with a Nikon Eclipse Ecliose NiU microscope (Nikon Instruments, Melville, NY) and digitized with SPOT software 5.0 (Diagnostic Instruments, Inc. USA).

### Tissue preparation, immunofluorescence staining, and image analysis

The 9-month-old mice were anesthetized by ketamine/xylazine (10/2 mg/ml, IP injection), and transcardially perfused with ice-cold PBS, followed by 4% paraformaldehyde in PBS. Dissected brains were harvested and post fixed with 4% paraformaldehyde at 4 °C for 24 h, and then dehydrated in 30% sucrose in PBS and stored at 4 °C. Fixed brains were sectioned in 10 μm with a cryostat.

For immunofluorescent staining as described previously [51], sections were incubated with the following primary antibody at 4 °C overnight: rabbit anti-Iba1(1:200, Wako, Japan), goat anti-Iba1 (1:200, Novus Biologicals, CO, USA) ), rabbit anti-AdipoR1 (1:100, Abcam, Cambridge, MA, USA), rabbit anti-AdipoR2 (1:100, Boster Biological Technology, USA), rabbit anti-TNFα (1:100, Cell Signaling Tech. Inc., USA), mouse anti-IL-1β (1:100, Cell Signaling Tech. Inc., USA), and mouse anti-glial fibrillary acidic protein (GFAP, 1:200, Santa Cruz) followed by appropriate Alexa-Fluor-conjugated secondary antibody (Thermo Fisher Scientific, USA) at RT for 1 h. Sections were mounted with slow fade® anti-fade DAPI reagent (Lifetech, USA). In each treatment group, a total of 24 sections (6 sections/mouse; *n* = 4) were acquired with a Nikon Eclipse NiU microscope (Nikon Instruments, Melville, NY) and digitized with SPOT software 5.0 (Diagnostic Instruments, Inc. USA) in identical settings to determine the degree of overlap between AdipoR1, AdipoR2, TNFα or IL-1β immunoreactive cells, and Iba1-expressing cells in the cortex and hippocampus. Analysis of the fluorescent intensities and the number of labeled TNFα or IL-1β-positive microglia cells were quantified with ImageJ software (Wayne Rasband NIH, USA).

For immunohistochemistry, mice at 9 months old were anesthetized with ketamine/xylazine (10/2 mg/ml, IP injection). After transcardially perfused with PBS and 4% paraformaldehyde, dissected brains were post fixed in 4% paraformaldehyde for 24 h at 4 °C. Fixed brains were incubated in gradient ethanol for dehydration followed by xylene before embedding in paraffin wax. Tissue sections (10 μm thick) were obtained by using a rotary microtome. Tissue sections were rehydrated by graded ethanol to water before antigen retrieval. Hydrogen peroxide solution was used to inactivate endogenous peroxidase. Sections were incubated with a primary antibody (rabbit anti-p-AMPK^T172^, 1:100, Cell Signaling Tech. Inc., USA) at 4 °C overnight, followed by incubation with HRP-conjugated secondary antibody (goat anti-rabbit, 1:200; Dako, Glostrup, Denmark) at RT for 1 h. Sections were developed by brown color staining and counterstained with hematoxylin.

### Quantification of amyloid plaques and the number of microglia around plaques

Each cryosection (40 μm thick) was stained with 0.01% thioflavin-S (Sigma-Aldrich) followed by immunostaining with anti-Iba1 (Wako Chemicals). The quantitative images of amyloid plaque were selected in the similar location in the cortex (10 sections/mouse; *n* = 2) and acquired with a water-immersion objective lens (× 10 or × 20) of Nikon Eclipse NiU microscope (× 10 microscope eyepieces, Nikon Instruments, Melville, NY). The number of amyloid plaques was counted with ImageJ cell counter plugin. To determine the number of microglia around plaques, the quantitative images of amyloid plaque were randomly selected for their similar size in the cortex (*n* = 80 plaques from 2 mice per genotype) using a water-immersion objective lens (× 20 or × 40) of Nikon Eclipse NiU microscope (× 10 microscope eyepieces, Nikon Instruments, Melville, NY). The cell bodies of microglia within a 25-μm radius circular area from the plaque edge were quantified with ImageJ software (Wayne Rasband NIH, USA).

### BV2 conditioned medium (CM)

BV2 cells were seeded in a 6-well plate to 80% confluent. The cells were pretreated with APN or Compound C (10 μM) for 2 h followed by AβO treatment for 24 h in serum-free culture medium. After incubation, the supernatant (CM) was collected and filtered with a 0.22-μm sterile filter (Acrodisc®; Pall Corporation) to remove cells and cell debris. For the control group, BV2 cells were incubated for with medium only for 24 h. For activation of HT-22 cells, the cells were seeded in 96-well plates (1.5 × 10^4^/well) in serum-free culture medium and then treated with BV2 CM for 24 h.

### Transwell assay

HT-22 cells (1 × 10^5^/well) were seeded in 24-well plate in serum-free culture medium. BV2 cells (1.5 × 10^4^/well) were seeded in the inserts (Cell Culture Inserts for 24-well plates, 0.4-μm pore size, Polyester (PET) Membrane, Corning Costar Corp, USA) and directly placed above the HT-22 culture. BV2 cells were pretreated with APN or Compound C for 2 h followed by AβO treatment for 24 h. Then, the viability of HT-22 cells in the lower compartment was determined by MTT assay.

### MTT assay and cell viability of HT-22 cells

Cell viability was evaluated with 3-(4, 5-dimethylthiazolyl-2)-2, 5-diphenyltetrazolium bromide (MTT). HT-22 cells were incubated with MTT solution at a concentration of 5 mg/ml in a humidified incubator at 37 °C for 1 h. Afterwards, the supernatants were removed and then 200 μl of dimethyl sulfoxide (DMSO) was added to detect formazan crystal developed in the viable cells. The absorbance of the solution in each well was determined at 570 nm using a CLARIO star microplate reader (BMG LABTECH, Germany) as described by the manufacturer. Cell viability was expressed as a percentage of viable cells obtained relative to that of controls.

### Statistical analyses

Statistical analyses were performed by Prism 6 (GraphPad Software, Inc., La Jolla, CA, USA). Quantitative data were expressed as mean ± standard errors of the mean (S.E.M.) and analyzed using one- and two-way ANOVA followed by post hoc comparison using Tukey’s test. The difference was considered to be statistically significant when *P* < 0.05.

## Results

### AdipoR1 and AdipoR2 were expressed in BV2 cells and microglia cells of mice

**Fig. 1:**
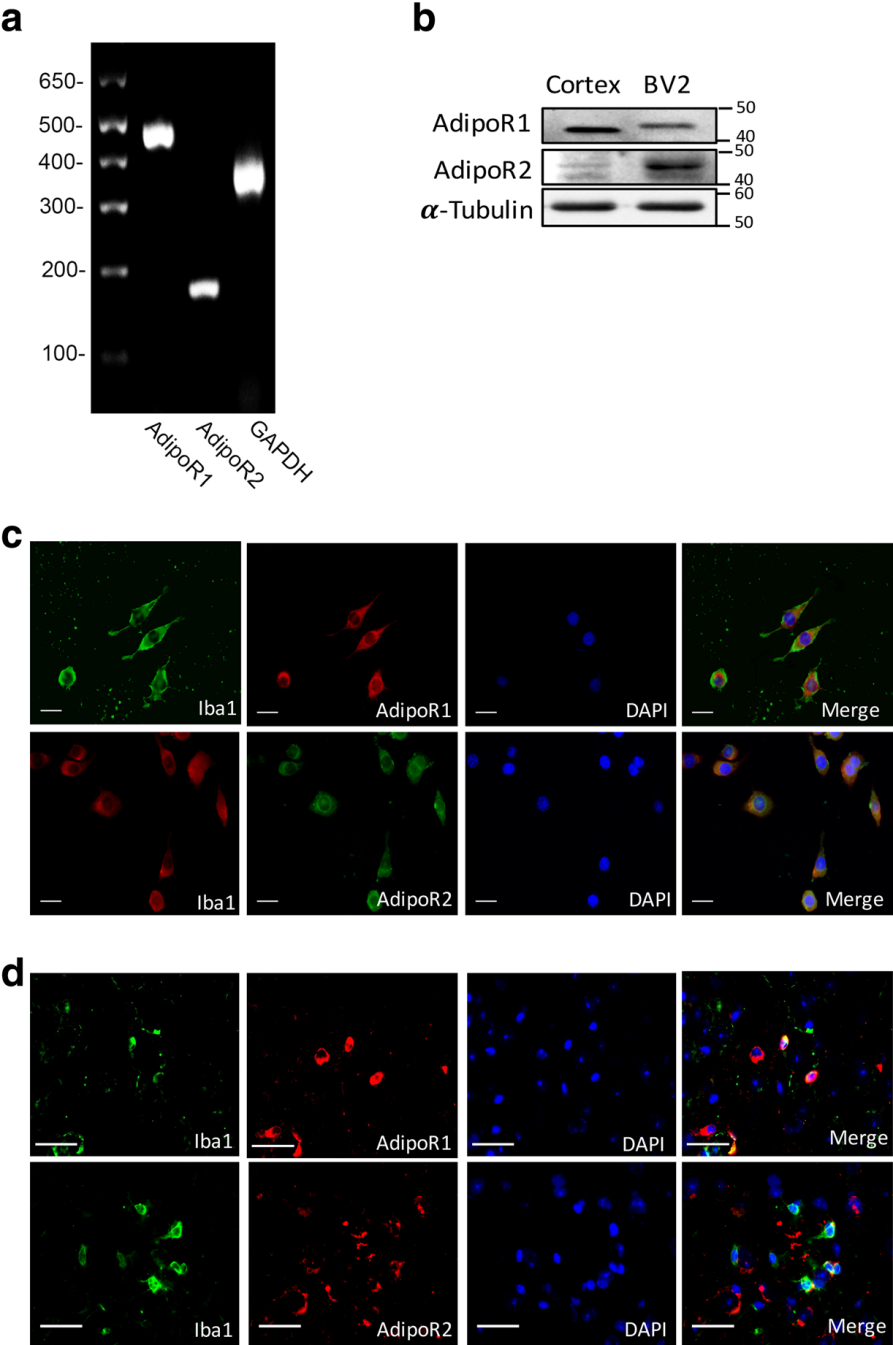
Expression of AdipoR1 and AdipoR2 in BV2 cells and microglia in mice brain. **a** RT-PCR analysis of AdipoR1 and AdipoR2 in BV2 cells. **b** Western blot analysis of AdipoR1 and AdipoR2 expression in BV2 cells. Expression of AdipoR1 and AdipoR2 from cerebral cortex homogenates was used as a positive control. α-Tubulin was used as a loading control. **c**, **d** Co-immunocytochemistry staining of microglia (Iba1) and AdipoR1 or AdipoR2 in BV2 cells and microglia in the cortex of WT mice. Scale bar 50 μm

Previous studies have shown that biological effects of APN were mediated through AdipoR1 and AdipoR2 [22]. In order to identify the expression of AdipoR1 and AdipoR2 in BV2 microglia cell line and microglia cell in vivo, we performed reverse transcriptional PCR and Western blot analysis to detect mRNA and proteins respectively. The results demonstrated that AdipoR1 and AdipoR2 were expressed in mouse microglial BV2 cells at both mRNA (Fig. 1a) and protein levels (Fig. 1b). Immunofluorescence staining showed that AdipoR1 and AdipoR2 were expressed in the cell body and processes of BV2 cells (Fig. 1c) and microglia cells in the cortex of WT mice (Fig. 1d).

### APN suppressed AβO-induced proinflammatory cytokine release in BV2 cells

**Fig. 2:**
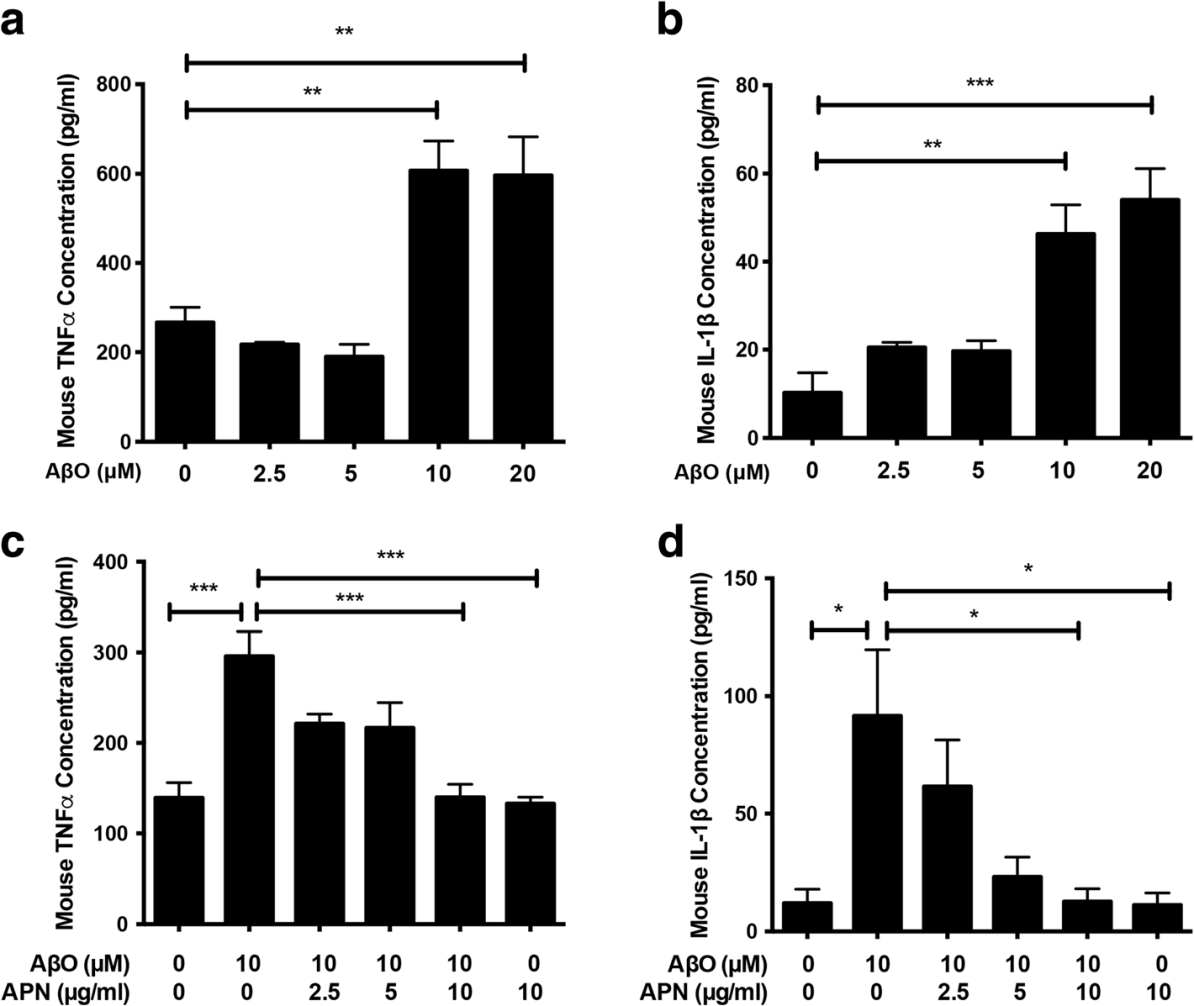
APN suppressed AβO-induced proinflammatory cytokines release in BV2 cells. **a**, **b** BV2 cells were treated with different concentrations of AβO (0, 2.5, 5, 10, and 20 μM) for 24 h, and then ELISA assays of TNFα and IL-1β were conducted. **c**, **d** Cells were pretreated with different concentrations of APN (0, 2.5, 5, and 10 μg/ml) for 2 h, followed by exposure of AβO (10 μM) for another 24 h, and then ELISA assays of TNFα and IL-1β were conducted. Data were presented as the mean ± SEM for at least three independent experiments, and each performed in duplicates (*n* = 3). One-way ANOVA with Tukey’s multiple comparison test revealed a difference between groups. *p < 0.05, **p < 0.01, ***p < 0.001

Next, we investigated the proinflammatory effect of AβO on BV2 cells. We found that exposure of AβO for 24 h induced the release of TNFα (Fig. 2a) and IL-1β (Fig. 2b) from BV2 cells in a concentration-dependent manner. Significant increase of TNFα and IL-1β levels was induced by AβO at a concentration of 10 μM. Thus, 10 μM was chosen as the concentration of AβO in all further experiments. To investigate if APN protects BV2 cells against AβO-induced proinflammatory cytokine release, BV2 cells were pretreated with APN for 2 h prior to exposure to AβO for 24 h. TNFα and IL-1β levels were markedly increased by AβO treatment, whereas APN inhibited the release of TNFα (Fig. 2c) and IL-1β (Fig. 2d) in a concentration-dependent manner, with a significant effect at the concentration of 10 μg/ml. Together, these data suggest that APN strongly inhibits AβO-induced proinflammatory cytokine expression in microglia cells.

The morphological transformation of microglia from ramified morphology to amoeboid shape was associated with inflammation and neurotoxicity [52]. The morphology of BV2 cells induced by AβO or APN was also examined. AβO-treated BV2 cells revealed an amoeboid shape with enlarged cell body, and extended processes were lost. Pretreatment with APN ameliorated the morphological changes of BV2 cells caused by AβO. The control and APN only-treated BV2 cells also showed a ramified morphology (Additional file 1).

### APN suppressed AβO-induced microglial proinflammatory cytokine release via AMPK-NF-κB signaling pathway

**Fig. 3:**
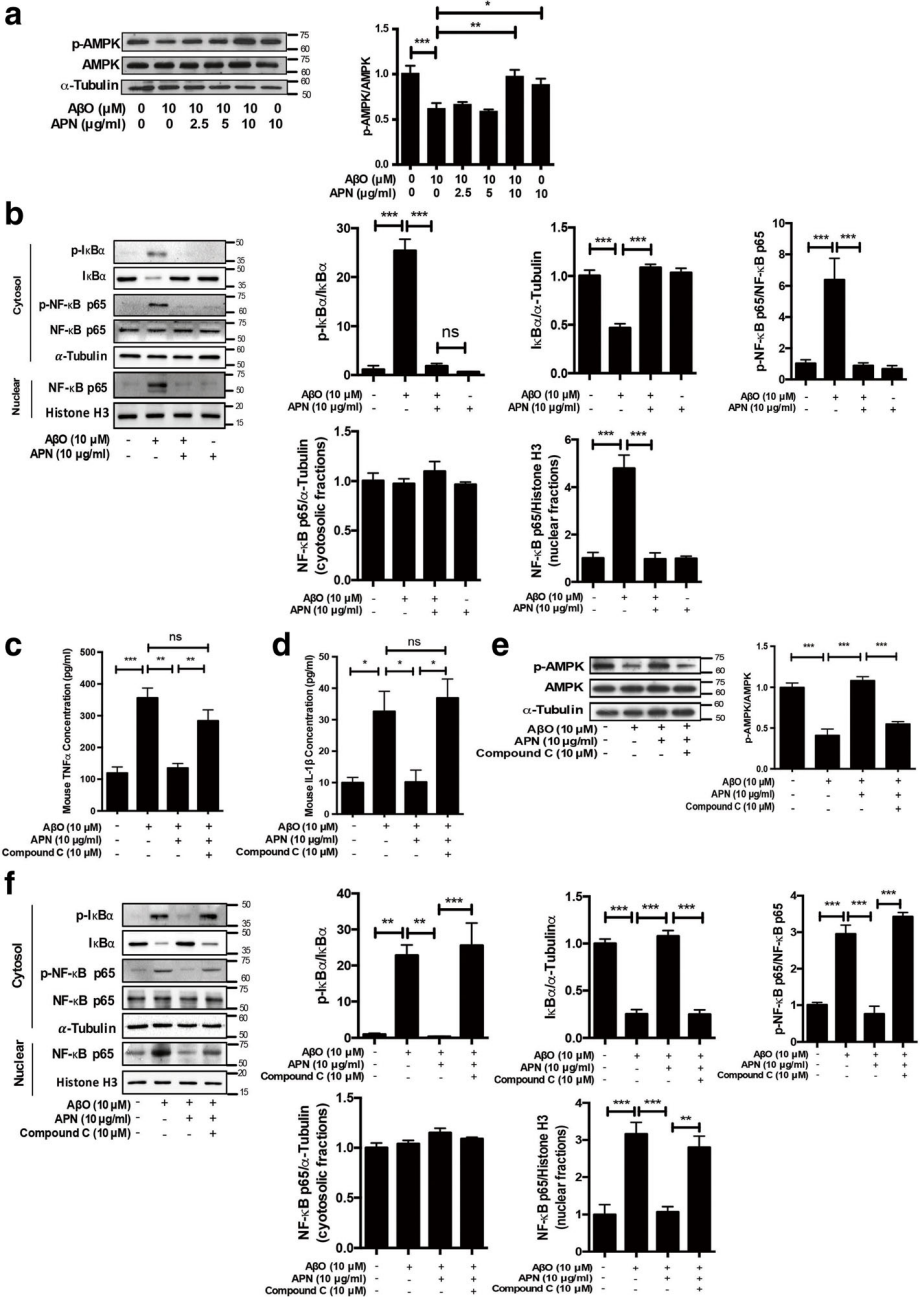
APN suppressed AβO-induced proinflammatory cytokine release in BV2 cells via AMPK-NF-κB signaling pathway. **a** Representative western blot analysis of AMPK phosphorylation. α-Tubulin immunoreactivity was used as a loading control (*n* = 6). **b** Representative western blot analysis of NF-κB activation in BV2 cells. The cytosolic fractions were prepared and analyzed with phosphorylated IκBα, total IκBα, phosphorylated NF-kB p65^S536^, and total NF-kB p65. The nuclear fractions were prepared and analyzed with total NF-kB p65. α-Tubulin immunoreactivity was used as a loading control in the cytosolic fraction, and histone H3 was used as the loading control in the nuclear fraction (*n* = 5). **c**, **d** BV2 cells were pretreated with AMPK inhibitor Compound C (10 μM) for 2 h, and then were treated with APN for 2 h, followed by exposure of AβO for 24 h. ELISA assays of TNFα and IL-1β were conducted (*n* = 3). **e** Representative western blot analysis of AMPK phosphorylation (*n* = 6). **f** Representative western blot analysis of NF-κB activation in BV2 cells (*n* = 5). Data were presented as the mean ± SEM. One-way ANOVA with Tukey’s multiple comparison test revealed a difference between groups. *p < 0.05, **p < 0.01, ***p < 0.001; ns, statistically not significant

5′AMP-activated protein kinase (AMPK) is a known energy metabolic sensor that is activated by phosphorylated AMPK^T172^ by LKB1 complex in response to an enhanced cellular AMP/ATP ratio [53]. Accumulating evidence has shown that APN mediates AMPK activation through AdipoR1 and AdipoR2 to facilitate metabolic processes in several target tissues [54]. AMPK, downstream mediator of APN, is involved in the inhibition of NF-κB activation and suppression of inflammation [55]. Therefore, we first analyzed phosphorylation of AMPK and NF-κB activation to understand whether AMPK pathway was involved in anti-inflammatory effect of APN on AβO-treated BV2 cells. Western blot analyses revealed that the amount of p-AMPK^T172^ was decreased in AβO-treated BV2 cells. In contrast, pretreatment with APN rescued the reduction of p-AMPK^T172^ upon AβO treatment (Fig. 3a). We next examined the effect of APN on the activation of NF-κB pathway in AβO-treated BV2 cells. Western blot analyses revealed that APN inhibited the increase of phosphorylated IκBα and the decrease of total IκBα induced by AβO. Consistent with this, the level of phosphorylated NF-κB p65^S536^, which can be induced by rapid degradation of IκBα in the cytoplasm, was markedly increased by AβO, whereas pretreatment with APN decreased phosphorylated NF-κB p65^S536^ upon AβO-treated BV2 cells. Nuclear accumulation of NF-κB p65 was increased in AβO-treated BV2 cells, whereas pretreatment with APN reduced nuclear NF-κB p65 level significantly (Fig. 3b). These data suggest that APN increases AMPK activation and reduces the translocation of NF-κB into the nuclear of AβO-treated BV2 cells. To further evaluate whether AMPK was responsible for the anti-inflammatory effect of APN on AβO-treated microglia cells, BV2 cells were pretreated with or without an AMPK inhibitor, Compound C, before the addition of APN and AβO. We found that APN decreased AβO-induced TNFα and IL-1β release in BV2 cells that were remarkably blocked upon pretreatment with Compound C (Fig. 3c, d). In addition, pretreatment with Compound C exhibited obvious conversion effects of APN on p-AMPK^T172^ and nuclear translocation of NF-κB in AβO-treated BV2 cells (Fig. 3e, g). Together, these data suggest that APN inhibits AβO-induced proinflammatory cytokines via the AMPK-NF-κB signaling pathway in microglia cells.

### APN suppressed AβO-induced microglial proinflammatory cytokine release via AdipoR1

**Fig. 4:**
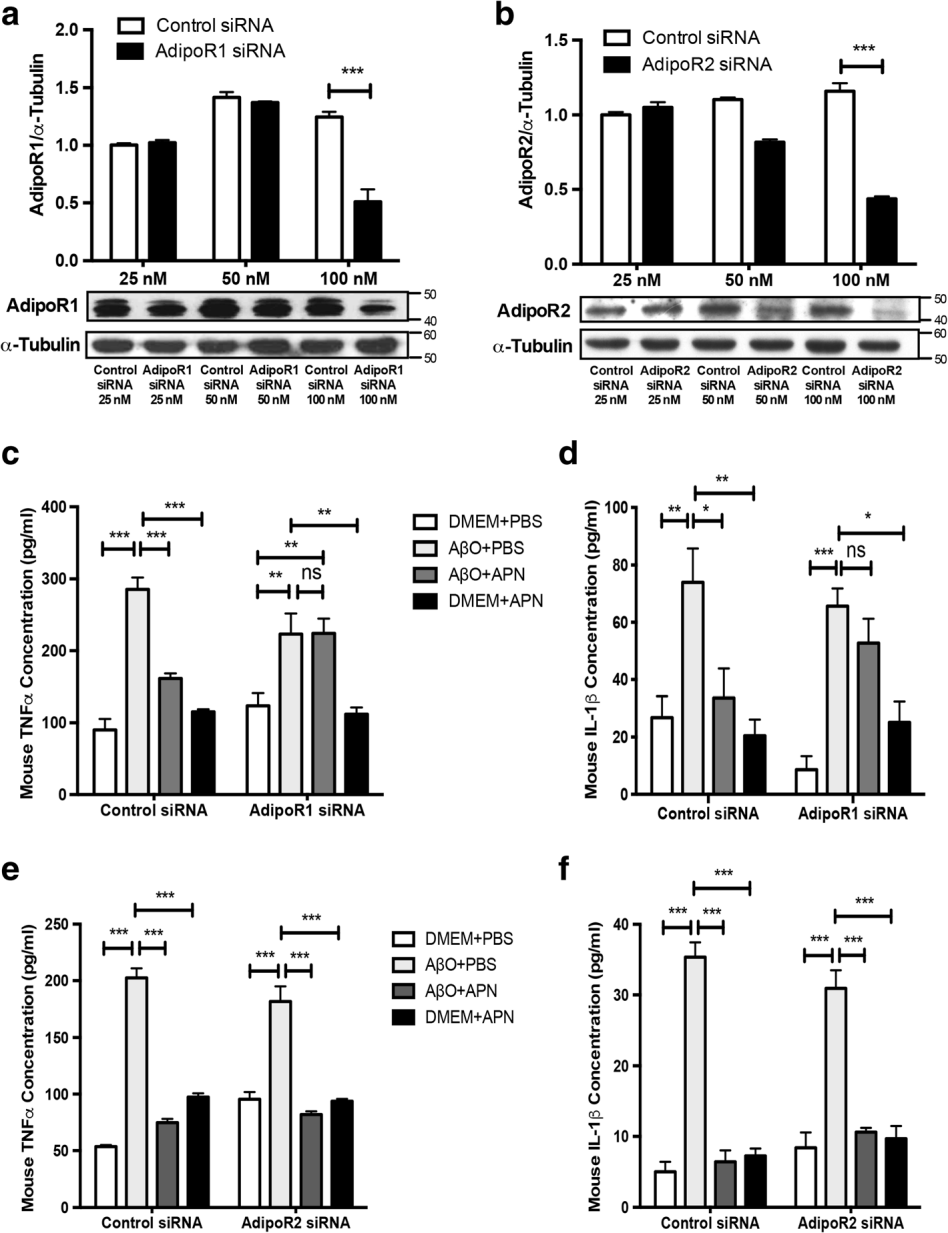
APN suppressed AβO-induced proinflammatory cytokine release in BV2 cells via AdipoR1. **a**, **b** Representative Western blot analysis of AdipoR1 and AdipoR2. BV2 cells were transfected with control siRNA, AdipoR1 siRNA, or AdipoR2 siRNA in a concentration-dependent manner (25, 50, 100 nM). **c**, **d** ELISA assays of TNFα and IL-1β were conducted after knockdown of AdipoR1. **e**, **f** ELISA assays of TNFα and IL-1β were conducted after knockdown of AdipoR2. Data were presented as the mean ± SEM for at least three independent experiments, and each performed in duplicates (*n* = 3). Two-way ANOVA with Tukey’s multiple comparison test revealed a difference between groups. *p < 0.05, **p < 0.01, ***p < 0.001; ns, statistically not significant

To investigate the roles of AdipoR1 and AdipoR2 in regulating the anti-inflammatory effect of APN on AβO-treated BV2 cells, we used siRNA to knock down AdipoR1 or AdipoR2. Western blot analyses showed that AdipoR1 and AdipoR2 expression were all significantly inhibited by siRNA at the dose of 100 nM (Fig. 4a, b). The ability of APN to suppress the release of proinflammatory cytokines TNFα and IL-1β in AβO-treated BV2 cells was abolished in AdipoR1 siRNA-transfected cells (Fig. 4c, d), but not in AdipoR2 siRNA-transfected BV2 cells (Fig. 4e, f). These data indicate that APN suppressed AβO-induced microglial proinflammatory cytokine release via AdipoR1.

### Anti-inflammatory effect of APN on AβO-exposed BV2 microglia cells protected HT-22 neuronal cells from cytotoxicity

**Fig. 5:**
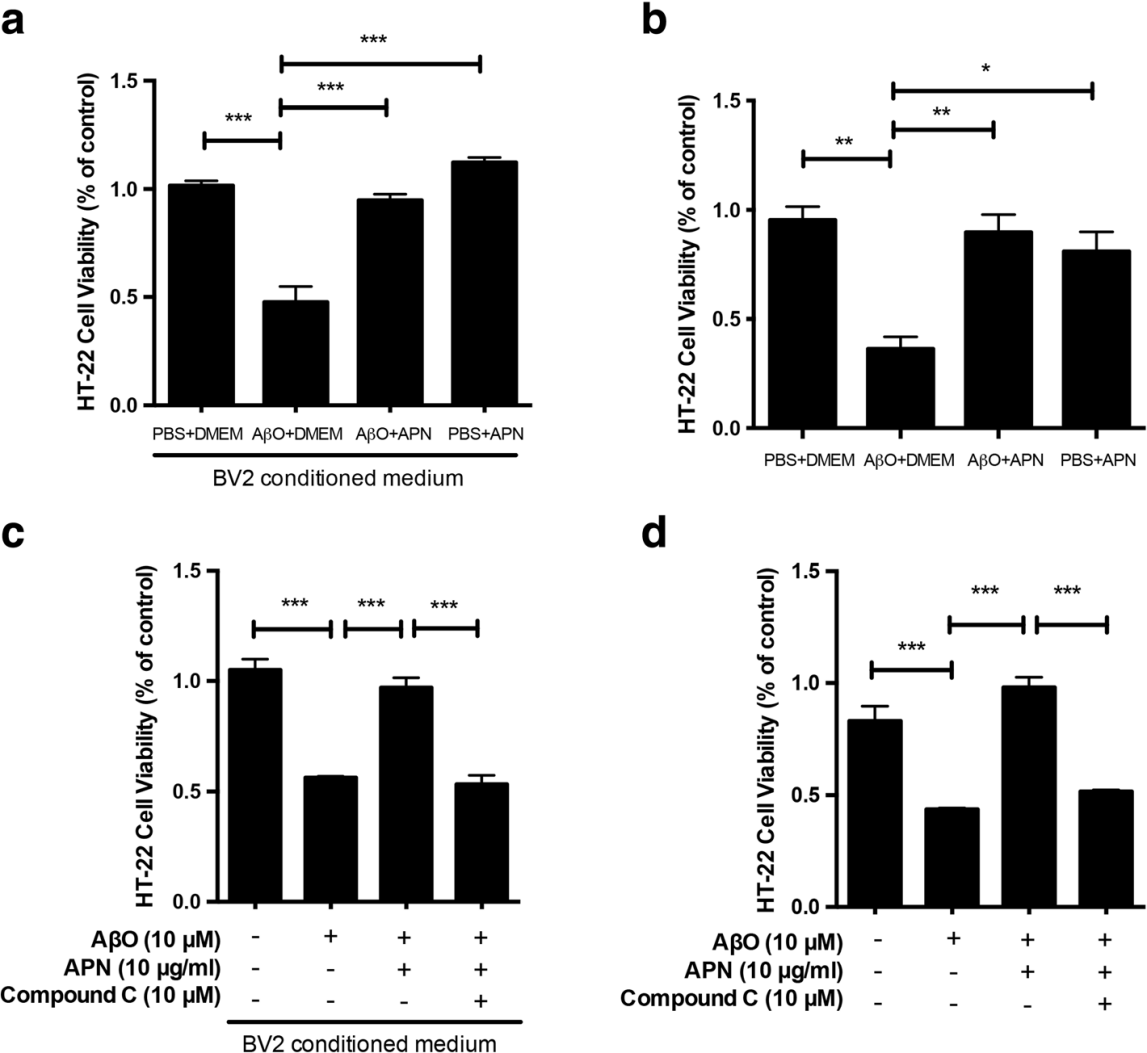
Anti-inflammatory effect of APN on AβO-treated BV2 cells protected HT-22 neuronal cells from cytotoxicity. **a** HT-22 cells were incubated for 24 h with conditioned medium derived from cultures of BV2 cells exposed by AβO with or without pre-treatment with APN. Medium from cultures of untreated microglia served as a control. **b** HT-22 cells were co-cultured with AβO-exposed BV2 cells with or without pre-treatment with APN for 24 h in a transwell system. HT-22 cells co-cultured with untreated BV2 cells served as a control. **c** HT-22 cells were incubated for 24 h with conditioned medium derived from cultures of AβO-exposed BV2 cells with pre-treatment with APN and Compound C. **d** HT-22 cells were co-cultured with AβO-exposed BV2 cells with pre-treatment with APN and Compound C in a transwell system. Cell viability was evaluated by the MTT assay. Data were presented as the mean ± SEM for at least three independent experiments, and each performed in triplicates (*n* = 3). One-way ANOVA with Tukey’s multiple comparison test revealed a difference between groups. *p < 0.05, **p < 0.01, ***p < 0.001

It has been demonstrated that activated microglia release toxic agents with neurotoxic effects which correlate with the onset and progression of neurodegenerative disease [56, 57]. Since we found that APN inhibited AβO-induced proinflammatory cytokine release in microglia, we next determined whether APN could protect against neuronal toxicity induced by AβO-activated microglia. We used an MTT assay to examine the effect of APN on AβO-induced toxicity from microglia to a hippocampal cell line, HT-22 neuronal cells. We found that when HT-22 cells were treated with conditioned medium from AβO-treated BV2 cells, there was a significant decrease in cell viability. However, when the cells were treated with conditioned medium from BV2 cells that had been incubated with APN and AβO, the cell viability was close to that of the control group (Fig. 5a). Hence, we suggest that APN protects HT-22 neuronal cells against indirect cytotoxicity induced by AβO-activated microglia. Next, we used a transwell co-culture system that allows diffusion of soluble molecules between BV2 cells and HT-22 cells without direct cell–cell contact. In accordance with previous findings, AβO-activated microglia reduced the viability of HT-22 neuronal cells, compared to the viability of HT-22 neuronal cells that co-cultured with vehicle-treated BV2 microglia cells. The viability of HT-22 neuronal cells co-cultured with BV2 microglia cells treated with AβO and APN was significantly increased, compared to those in co-culture with BV2 cells treated with AβO only (Fig. 5b). As Aβ is toxic to neurons and also impaired mitochondrial respiratory complex functions [53], we conducted the experiment to determine whether the decrease of the viability of HT-22 neuronal cells is due to the AβO or neurotoxicity of AβO-treated BV2 cells. We found that the viability of HT-22 cell that co-cultured with AβO-treated BV2 cells was decreased more than the viability of HT-22 cell treated with AβO without co-cultured BV2 cells (Additional file 2). Finally, we pretreated BV2 cells with Compound C to test whether AMPK played an essential role in the interactions between activated microglia and neurons. The presence of Compound C in the conditioned medium blocked the protective effect of APN on viability HT-22 neuronal cells (Fig. 5c). Co-culture of HT-22 neuronal cells with BV2 microglia cells pretreated with Compound C yielded similar results (Fig. 5d). Together, these data suggest that APN inhibits AβO-induced microglial cytotoxicity to HT-22 neuronal cells via AMPK activation.

### APN deficiency increases proinflammatory cytokine expression in the cortex and hippocampus in 5xFAD mice

**Fig. 6:**
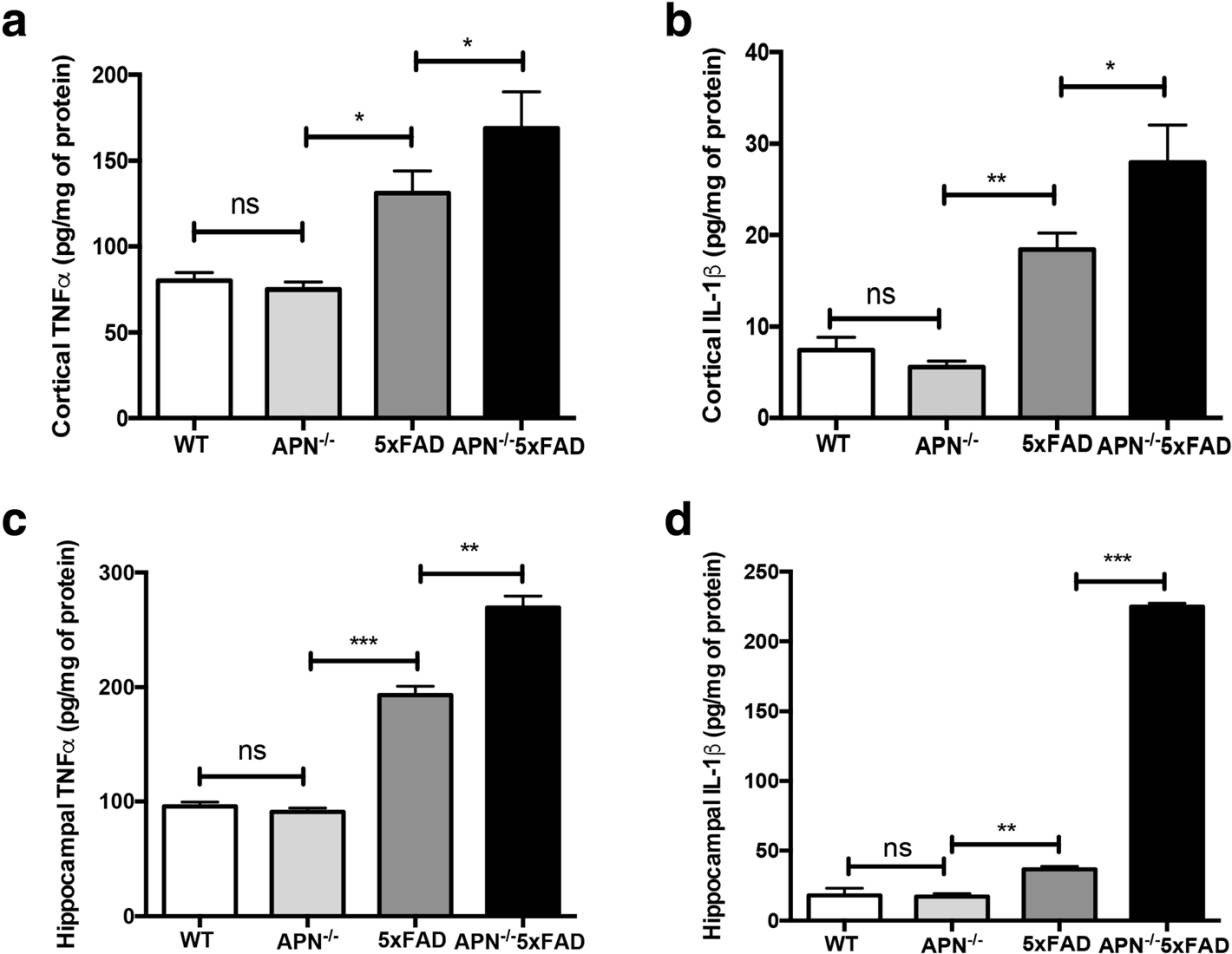
Proinflammatory cytokines were increased in the cortex and hippocampus of 5xFAD mice and APN^−/−^5xFAD mice. ELISA assays of TNFα and IL-1β in cortical (**a**, **b**) and hippocampal (**c**, **d**) homogenates of WT mice, APN^−/−^ mice, 5xFAD mice, and APN^−/−^5xFAD mice at 9 months old. Data were presented as the mean ± SEM for WT mice (*n* = 5–6), 5xFAD mice (*n* = 6), APN^−/−^ mice (*n* = 6), and APN^−/−^5xFAD mice (*n* = 6). One-way ANOVA with Tukey’s multiple comparison test revealed a difference between groups. *p < 0.05, **p < 0.01, ***p < 0.001; ns, statistically not significant

It has been well recognized that activated microglia induces neuroinflammation by generating excessive proinflammatory cytokines, which contributes to AD pathogenesis [13]. Recent studies have shown that APN inhibits neuroinflammatory responses in vivo [46, 48]. To examine whether APN deficiency increased neuroinflammation in a mouse model of AD, we generated APN-deficient AD mice (APN^−/−^5xFAD) by crossing APN knock-out mice (APN^−/−^) with 5xFAD mice (APN^−/−^5xFAD). We measured levels of the proinflammatory cytokines TNFα and IL-1β in the cortical and hippocampal fractions of wild type mice (WT), APN^−/−^ mice, 5xFAD mice, and APN^−/−^5xFAD mice at 9 months old. We found that TNFα and IL-1β levels were both increased in the cortex (Fig. 6a, b) and hippocampus (Fig. 6c, d) of 5xFAD mice and APN^−/−^5xFAD mice, compared with those of WT mice. In addition, TNFα and IL-1β levels were both higher in APN^−/−^5xFAD mice compared with those of 5xFAD mice. However, there is no difference of TNFα and IL-1β levels between WT mice and APN^−/−^ mice at 9 months old. These data suggest that APN deficiency leads to more intense neuroinflammation in AD.

### APN deficiency exacerbates microglia activation and proinflammatory cytokine expression in 5xFAD mice

**Fig. 7:**
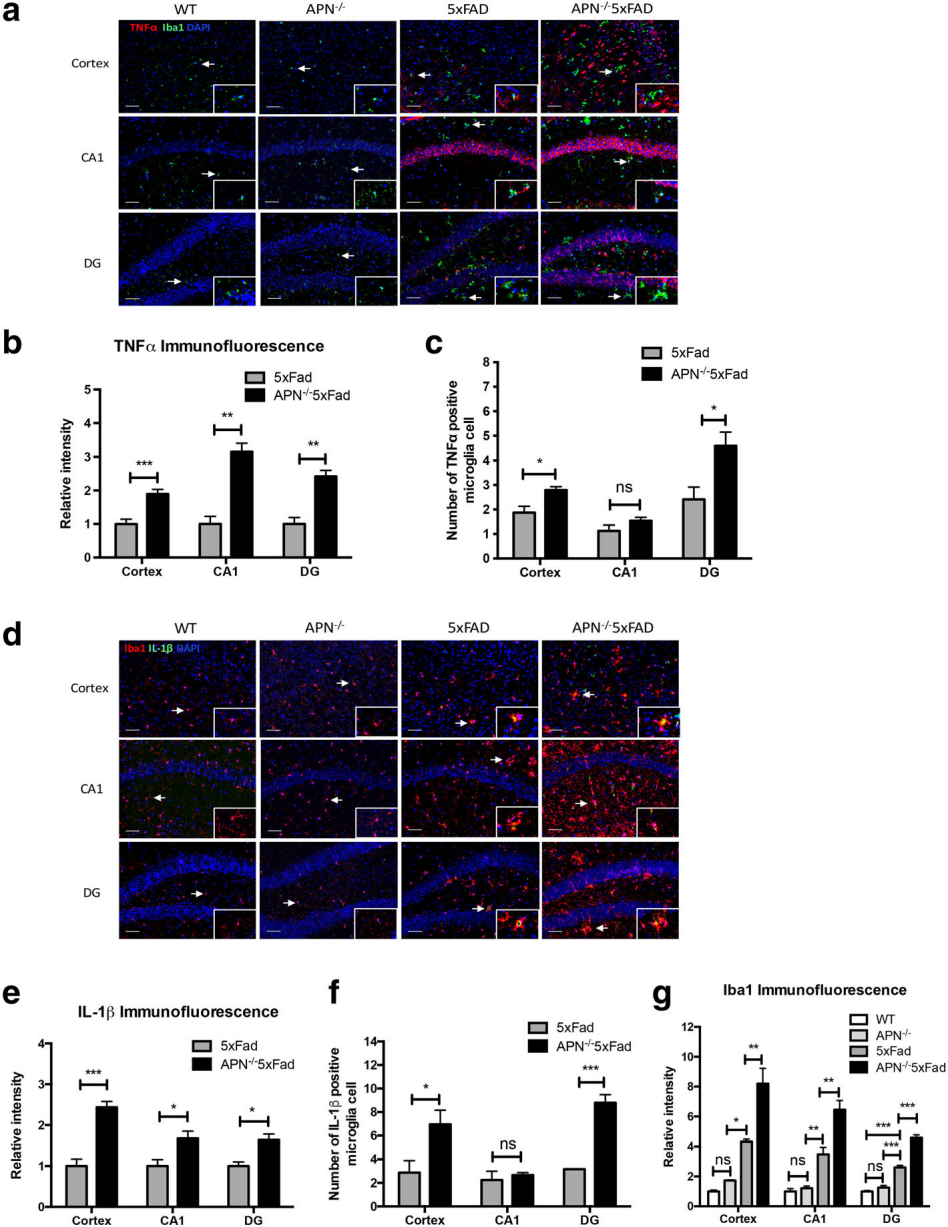
Microglia activation associated with proinflammatory cytokines was increased in 5xFAD mice and APN^−/−^5xFAD mice. **a** Representative images of double immunofluorescence staining for TNFα (red) with Iba1 (green) in the cortex, hippocampus CA1, and dentate gyrus (DG) of WT mice, APN^−/−^ mice, 5xFAD mice, and APN^−/−^5xFAD mice at 9 months old. Scale bar 50 μm. **b** Quantification of TNFα fluorescence intensity in the cortex, hippocampus CA1, and DG of WT mice, APN^−/−^ mice, 5xFAD mice, and APN^−/−^5xFAD mice (*n* = 4). **c** Quantification of the number of TNFα-positive microglia in the cortex, hippocampus CA1, and DG of WT mice, APN^−/−^ mice, 5xFAD mice, and APN^−/−^5xFAD mice (*n* = 4). **d** Representative images of double immunofluorescence staining for IL-1β (green) with Iba1 (red) in the cortex, hippocampus CA1, and DG of WT mice, APN^−/−^ mice, 5xFAD mice, and APN^−/−^5xFAD mice at 9 months old. Scale bar 50 μm. **e** Quantification of IL-1β fluorescence intensity in the cortex, hippocampus CA1, and DG of WT mice, APN^−/−^ mice, 5xFAD mice, and APN^−/−^5xFAD mice (*n* = 4). **f** Quantification of the number of TNFα-positive microglia in the cortex, hippocampus CA1, and DG of WT mice, APN^−/−^ mice, 5xFAD mice, and APN^−/−^5xFAD mice (*n* = 4). **g** Quantification of Iba1 fluorescence intensity in the cortex, hippocampus CA1, and DG of WT mice, APN^−/−^ mice, 5xFAD mice, and APN^−/−^5xFAD mice. Right bottom corner represented a magnified image × 400. One-way ANOVA with Tukey’s multiple comparison test revealed a difference between groups. *p < 0.05, **p < 0.01, ***p < 0.001; ns, statistically not significant

To further determine whether APN deficiency exacerbates microglial activation and proinflammatory cytokine expression in APN^−/−^5xFAD mice, immunofluorescence staining experiments were conducted. In agreement with previous results, TNFα immunoreactivities were significantly increased in the cortex, hippocampus CA1, and dentate gyrus (DG) of APN^−/−^5xFAD mice, compared with that of the 5xFAD (Fig. 7a, b). Double immunofluorescence staining revealed co-localization of Iba1 and TNFα indicating an increased expression of TNFα in microglia were observed in the DG of APN^−/−^5xFAD mice (Fig. 7c). IL-1β immunoreactivities were also increased in the cortex, hippocampus CA1, and DG of APN^−/−^5xFAD mice, compared with that in the 5xFAD (Fig. 7d, e). Co-localization of Iba1 and IL-1β showed that expression of IL-1β in microglia were increased in the cortex and DG of APN^−/−^5xFAD mice (Fig. 7f). In addition, we found that Iba1 immunoreactivities were increased in the cortex, hippocampus CA1, and DG in APN^−/−^5xFAD mice, compared with those in WT mice, APN^−/−^ mice, and 5xFAD mice (Fig. 7g). We also evaluated astrogliosis which was another pathological hallmark of AD between the mice. We found that APN^−/−^5xFAD mice also showed stronger GFAP immunoreactivities in the cortex and hippocampus, compared with that of WT mice, APN^−/−^ mice, and 5xFAD mice suggestive of increased astrogliosis (Additional file 3). Moreover, we also examined the level of p-AMPK^T172^ between APN^−/−^5xFAD mice and 5xFAD mice. We found that a lowered level of p-AMPK^T172^ was observed in the cortex of APN^−/−^5xFAD mice, compared with that of 5xFAD mice (Additional file 4). Finally, we found that there was no difference of amyloid deposition in the cortex between 5xFAD mice and APN^−/−^5xFAD mice. However, we observed that the size of amyloid plaques was different between these mice. Then, we categorized amyloid plaque on the basis of size (small plaque radius < 10 μm; 10 μm < medium plaque radius < 15 μm; large plaque radius > 15 μm) in the cortex. We found that APN^−/−^5xFAD mice showed a lower proportion of small amyloid plaques, but a higher proportion of large amyloid plaques, compared with those in 5xFAD mice. We also found that the number of microglia within a predefined area of 25-μm radius around small plaques and medium plaques was decreased in the APN^−/−^5xFAD mice, compared with that in the 5xFAD mice (Additional file 5). These data suggest that APN deficiency results in increased activation and neuroinflammatory response of microglia and decreased clustering microglia around Aβ plaques in AD.

## Discussion

In the current study, we showed that APN suppressed microglia-mediated neuroinflammation induced by AβO. APN inhibited the proinflammatory cytokines TNFα and IL-1β in AβO-exposed BV2 cells. This anti-inflammatory effect in microglia is regulated through AdipoR1-AMPK-NF-κB signaling pathway. Moreover, pretreatment of AβO-exposed BV2 cells with APN protected HT-22 neuronal cells against cytotoxicity induced by conditioned medium of AβO-exposed BV2 cells. Finally, APN deficiency increased microglia activation and proinflammatory cytokine levels in 5xFAD mice.

Our results were consistent with two recent studies which report that APN suppressed proinflammatory responses of microglia. Acrp30, a globular form of APN, was showed to reduce proinflammatory response and promote anti-inflammatory response in microglia exposed to AβO through peroxisome proliferator-activated receptor (PPAR)-γ signaling [58]. Nicolas et al. reported that ICV injection of globular APN reduced proinflammatory response of microglia stimulated by LPS via AdipoR1-NF-κB signaling in vivo [46]. Our findings showed that full-length APN also exerted anti-inflammatory effects by reducing synthesis and secretion of TNFα and IL-1β from microglia exposed to AβO via AdipoR1-AMPK-NF-κB signaling.

AMPK acts as an energy sensor and plays an important role in the regulation of energy metabolic homeostasis. In response to low AMP concentration, AMPK is activated by phosphorylation of α subunit at Thr172 via upstream kinases to increase cellular ADP to ATP and AMP to ATP ratios [59]. AMPK activated by APN is mediated by adaptor protein APPL1 binding to AdipoR1 or AdipoR2 [53, 60]. The role of AMPK in AD is not fully understood. Substantial studies showed that Aβ impaired mitochondrial respiratory complex functions and evoked AMPK activation in AD [53]. In contrast, a study demonstrated that oligomeric Aβ_42_ impaired AMPK phosphorylation to activate GSK3β and induce tau hyperphosphorylation in a fly model of AD [61]. Our results showed that pretreatment with APN prevented the decrease of phosphorylated AMPK in AβO-exposed microglia. Moreover, Compound C (C24H25N5O; 6-[4-(2-piperidin-1-ylethoxy) phenyl]-3-pyridin-4-ylpyrazolo [1, 5-a] pyrimidine), also called dorsomorphin, has been widely used as a selective and reversible AMPK inhibitor [62]. In this study, we found that Compound C blocked the effects of APN on AMPK activation in AβO-exposed microglia. These results were consistent with the findings of a recent study showing that exposure to Aβ or transfection with APPswe/ind in SH-SY5Y cells induced inhibition of AMPK activation, and this inhibitory effect could be prevented by osmotin, a plant protein homolog of mammalian adiponectin [63]. It had been shown that exposure to LPS in macrophages reduced AMPK activation, whereas exposure to anti-inflammatory cytokines promoted AMPK activation [64]. We believed that AMPK was a potential mediator of microglia inflammatory status. Based on the results, we speculated that persistent AβO stimulation resulted in AMPK dephosphorylation which might promote microglia to polarize to a pro-inflammatory phenotype, and APN induced AMPK activation and protected microglia against such proinflammatory polarization.

Activation of NF-κB is critical in the transcription of genes that are involved in the inflammatory response [65]. Briefly, in resting cells, NF-κB is retained in the cytoplasm by interacting directly with IκBα, an inhibitor protein in the IκB family. Activation of the IκB kinase (IKK) by various extracellular and intracellular stimuli can mediate the phosphorylation of IκBα and subsequently triggers rapid degradation of IκBα. This induces the phosphorylation of NF-κB p65 at Ser536 and allows liberated NF-κB to translocate into the nucleus, where NF-κB can regulate gene transcription [66]. In our study, we found that APN inhibited NF-κB translocation into the nucleus in AβO-treated BV2 cells. Moreover, the level of NF-κB p65 in nuclear was decreased by APN in AβO-treated BV2 cells. Phosphorylation at specific sites of NF-κB p65 regulates the transcriptional activity of p65 in the nucleus. It was reported that phosphorylation of p65^S276^ was required for the interaction of p65 with target gene and thereby promoted p65 transcriptional activity, while phosphorylation of p65^S536^ increased proteasomal degradation and nuclear export of p65 [67]. Our results also showed that APN inhibited nuclear translocation of NF-κB induced by AβO in microglia and the inhibition was dependent on AMPK activation. It had been reported that Acrp30 affected AβO-treated microglia through PPAR-γ signaling and NF-κB activation [58]. AMPK repressed NF-κB activation through multiple signaling pathways including silent information regulator 1 (SIRT1) [68], Forkhead Box O (FoxO) family [69], and peroxisome proliferator-activated receptor γ co-activator 1-α (PGC-1α) signaling [70, 71], which could subsequently inhibit the production of proinflammatory cytokines [55]. Our previous study reported that APN protected SH-SY5Y neuroblastoma cells overexpressing AβO from cytotoxicity under oxidative stress and it was through APPL1-mediated AMPK activation that suppressed the NF-κB activation [47].

AdipoR1 and AdipoR2 are two major receptors for APN. It has been reported that AdipoR1 and AdipoR2 had distinct binding affinities. AdipoR1 bound to gAd with high affinity, while AdipoR2 had high binding affinity to both globular and full-length APN [22]. Besides it, AdipoR1 and AdipoR2 had different functional signaling preferences. AdipoR1 activated AMPK, while AdipoR2 induced peroxisome proliferator-activated receptor-α signaling [54, 72, 73]. Their functions underlying neuroinflammation in AD were not clear. We found that the protective effects of APN on microglial inflammatory response induced by AβO were mediated by AdipoR1, but not AdipoR2. It seemed that anti-inflammatory effects of APN in microglia were not limited to the difference of binding affinities. AdipoR1 mediated the effects of both gAPN and full-length APN in preventing LPS-induced proinflammatory cytokine release from microglia [46]. Administration of an agonist of AdipoR1 attenuated neuroinflammation after intracerebral hemorrhage in mice [74]. In addition, expression levels of AdipoR1 and AdipoR2 affected tissue-dependent functions of APN. Both tyrosine kinase receptor superfamilies and G-protein coupled receptors were able to form multimeric complexes (homomers and heteromers) that differentially regulated distinct signaling effectors when activated by a given ligand [75, 76]. AdipoR1 had been shown to form homodimers in various cell lines [77]. A recent study showed that AdipoR1 and AdipoR2 formed homo- and heteromeric complexes under resting conditions. Upon activation of APN, both homo- and heteromeric adiponectin receptor complexes dissociated and formation of heteromer induced a delay of AMPK activation, which suggested that decrease of AdipoR2 expression would increase the proportion of AdipoR1 homodimers and enhance the AdipoR1 signaling [78]. Further studies about the functions of AdipoR1 and AdipoR2 in microglia are necessary to clarify their role in neuroinflammation of AD.

It was notable that APN inhibited the release of TNFα and IL-1β levels from AβO-exposed BV2 cells. Moreover, APN prevented cytotoxicity of HT-22 neuronal cells that were co-cultured with AβO-exposed BV2 cells. TNFα was an important mediator of neuroinflammation that was widely studied in AD. TNFα antagonist prevented inhibition of long-term potentiation (LTP) induced by Aβ [79]. Also, TNFα signaling pathway was involved in AβO-mediated microglial activation-induced neuronal cell cycle events (CCEs), which was an indicator of neuronal distress. Genetic deficiency of TNFα in AD transgenic mice failed to induce neuronal CCEs [80]. I.C.V injection of AβO induced phosphorylation of double-stranded RNA-dependent protein kinase (PKR) and eukaryotic translation initiation factor 2α (eIF2α) which triggered a decrease of synaptophysin and PSD-95 levels and cognitive impairment in WT mice, but not in TNFR^−/−^ mice [81]. Furthermore, deletion of tumor necrosis factor type 1 death receptor (TNFR1) in APP23 transgenic mice inhibits Aβ generation and Aβ plaque formation, reduces microglia activation, and prevents memory deficits [82]. A prospective, single-center, and open-label clinical pilot study reveals that patients with mild-to-severe AD who received etanercept, a TNFα inhibitor, given by perispinal extrathecal administration for 6 months had improved cognitive performance [83]. IL-1β was also a key factor in regulating inflammatory response in AD. It has been reported that elevated levels of IL-1β were detected in patients with early-onset Alzheimer’s disease [84]. In addition, LPS and IL-1β stimulation in microglia increased phosphorylation of neuronal tau and reduced synaptophysin levels in cortical neurons and induced neuronal cell loss. These effects of IL-1β on tau and synaptophysin were mediated through activation of p38-MAPK [85]. Our findings demonstrated that APN protected hippocampal neurons against cytotoxicity upon exposure to conditioned medium of AβO-exposed microglial cells, which suggested that the protective effects were due to suppressed secretion of neurotoxic TNFα and IL-1β from microglia cells.

We previously reported that microglia activation and proinflammatory cytokines TNFα and IL-1β were increased in the cortex and hippocampus of aged APN-KO mice [48]. In the current study, our results showed that APN deficiency in AD mice aggravated microglia activation associated with higher levels of proinflammatory cytokines TNFα and IL-1β in the cortex and hippocampus compared to AD mice without APN deficiency. It has been reported that Aβ deposits were surrounded by activated microglia cells, and elevated IL-1β levels were found in the brain of 5xFAD mice at 10 weeks old [86]. Systemic inflammatory challenge by LPS induced expression of IL-1β in microglia of 5xFAD mice at 12 months old [87]. We found that IL-1β levels and IL-1β-expressing microglia were increased in the cortex and hippocampus in both 5xFAD and APN^−/−^5xFAD mice. Abundant IL-1β-expressing microglia cells and non-microglia cells were found in APN^−/−^5xFAD mice. In the brain, IL-1β was synthesized and released mainly by the microglia and astrocytes [88, 89], and AdipoR1 was found to be expressed in astrocyte [74]. We speculated that APN also exerts its role in regulating inflammatory cytokines in astrocyte. Interestingly, we found that TNFα was predominantly expressed in non-microglia cells rather than in microglia cell in both 5xFAD mice and APN^−/−^5xFAD mice. APN^−/−^5xFAD mice had much more expression of TNFα than 5xFAD mice. We could not find direct evidence to show whether TNFα originated from neurons themselves or glia cells, but abundant studies have shown that the expression of TNFα was increased in neurons, microglia, reactive astrocytes, and epithelia cells upon brain injuries and chronic disorders [90]. In addition, we could not exclude the possibility that the source of TNFα was from the periphery. It has been reported that transport of TNFα could cross the intact blood–brain barrier (BBB) via both TNFα receptors [91]. Moreover, since the permeability of the BBB was increased during AD pathogenesis, peripheral immune cells might infiltrate into the brain parenchyma and produced TNFα [92, 93]. It had been suggested that APN exerted a protective role against BBB break down in AD. APN decreased secretion of proinflammatory cytokine IL-6 from brain endothelial cells, and other proinflammatory cytokines also had a decreased trend upon APN treatment [94]. Acrp30 also attenuated the tight junction disruption and reduced the proinflammatory cytokines in Aβ-exposed brain endothelial cells [95]. We speculated that APN deficiency would promote the BBB disruption in AD and aggravate neuroinflammation in AD. It was worth mentioning that expression of TNFα and IL-1β were not increased in APN^−/−^mice at 9 months old compared to wild-type mice, but we found cerebral IL-1β and TNFα levels were significantly increased at 18 months old in our previous study [48].

Microglial activation is associated with amyloidosis not only in transgenic mouse models of AD [96], but also in human AD [97]. In our study, APN deficiency induced a decrease of microglia clustering around small and medium fibrillar amyloid plaques, along with a significant increase of large amyloid plaque in 5xFAD mice. The function of plaque-associated microglia was not fully understood. Some study revealed that microglia barrier around the plaque would promote uptake of Aβ peptide and prevent additional fibrillization and outward plaque expansion, which exerted a neuroprotective role [98, 99]. Interestingly, in our study, we demonstrated widespread microglia activation but reduction of plaque-associated microglia in APN^−/−^5xFAD mice. One possibility was that APN deficiency in 5xFAD mice induced microglia into a dysfunctional state. We observed a large number of microglia cell display abnormal morphological features with shortened or gnarled processes and dystrophic spheroid formation, which might affect motility of microglia process contacting with the plaque and phagocytic ability of microglia [100, 101]. The mechanism underlying the effects of APN on microglia reaction to amyloid plaques and their involvement in amyloid plaque pathogenesis need to be further determined.

Some limitations should be noted in our study. Firstly, it was reported that adiponectin receptors were expressed in the hippocampus in mice [102]. Using the transwell co-culture system, we could not completely avoid potential effects of APN on HT-22 hippocampal cells which might be expressed adiponectin receptors. Secondly, BV2 cells did not model primary microglia completely in assessing the expression of inflammatory cytokines [103]. Thirdly, gene expressions in human microglia were environment-sensitive and altered significantly [104]. The effect of APN on neuroinflammation in the animal model and human patients with AD should be further studied.

## Conclusion

To conclude, we found that APN attenuated the inflammatory response of AβO-activated microglia via AdipoR1-AMPK-NF-κB signaling pathway, and APN deficiency enhanced neuroinflammation in the 5xFAD mouse model of AD. This anti-inflammatory effect of APN on AβO-exposed microglia might protect neurons against cytotoxicity in AD. The study provides evidence that APN may be a potential novel therapeutic agent in AD.

## Additional files

Additional file 1: APN treatment induced morphological changes of AβO-treated BV2 cells. Representative images were depicting morphology of BV2 cells pretreated with APN (10 μg/ml) for 2 h prior to incubation with AβO (10 μM) for an additional 24 h. Photomicrographs were taken directly from culture plates by phase-contrast microscopy. At the quiescent state, the BV2 microglia showed the typical ramified shape. Incubation with AβO of BV2 cells revealed an amoeboid shape which cell body was enlarged and extended processes were lost. Pretreatment with APN converted the amoeboid morphology of AβO-stimulated BV2 cells to a ramified morphology. Three independent experiments were performed. Scale bar 200 μm. (TIF 1630 kb)

Additional file 2: BV2 microglia in co-culture system exacerbated neuronal loss under AβO exposure. HT-22 cells were treated with AβO with or without pre-treatment of APN, compared with HT-22 cells co-cultured with AβO-exposed BV2 cells in a transwell system. Data were presented as the mean ± SEM for at least three independent experiments, and each performed in triplicates (*n* = 3). One-way ANOVA with Tukey’s multiple comparison test revealed a difference between groups. *p < 0.05. (TIF 199 kb)

Additional file 3: APN deficiency exacerbated astrogliosis in 5xFAD mice. (a) Representative images of GFAP immunoreactivity of astrocytes in the cortex and hippocampus of WT mice, APN^−/−^mice, 5xFAD mice, and APN^−/−^5xFAD mice at 9 months old. Scale bar 400 μm. (b) Quantification of GFAP fluorescence intensity WT mice, APN^−/−^mice, 5xFAD mice, and APN^−/−^5xFAD mice (*n* = 4). One-way ANOVA with Tukey’s multiple comparison test revealed the difference between groups. **p < 0.01, ***p < 0.001; ns, statistically not significant. (TIF 3235 kb)

Additional file 4: APN deficiency reduced the level of phosphorylated AMPK in 5xFAD mice. (a) Representative image of immunohistochemistry staining of p-AMPK^T172^ (black arrows) in the cortex of 5xFAD mice and APN^−/−^5xFAD mice at 9 months old. Scale bar 200 μm. (TIF 2716 kb)

Additional file 5: APN deficiency increased the amyloid plaque size and decreased microglia clustering around amyloid deposits in 5xFAD mice. (a) Representative images of thioflavin-S-labeled amyloid plaque (green) in the 5xFAD mice and APN^−/−^5xFAD mice. Scale bar 30 μm. (b) Quantification of the percentage of thioflavin-S-labeled amyloid plaques according to their plaque size (small plaque radius < 10 μm, arrowheads; 10 μm < medium plaque radius < 15 μm, dashed arrows; large plaque radius > 15 μm, solid arrows) in the cortex (10 sections/mouse; *n* = 2). (c) Representative images of Iba1-labeled microglia (red) surrounding different size of thioflavin-S-labeled amyloid plaque (green) in the 5xFAD mice and APN^−/−^5xFAD mice. Images on the right represented magnified portion of microglia clustering around amyloid plaque (× 400). Scale bar 30 μm. (d) Quantification of the number of Iba1-labeled microglia within a 25-μm radius from different size of plaque edge (*n* = 80 plaques from 2 mice per genotype). Data were presented as the mean ± SEM. Two-way ANOVA with Tukey’s multiple comparison test revealed a difference between groups. **p < 0.01, ***p < 0.001; ns, statistically not significant. (JPG 768 kb)

## Abbreviations

AD: Alzheimer’s disease
adipoR1: Adiponectin receptor 1
adipoR2: Adiponectin receptor 2
AMPK: 5′AMP-activated protein kinase
ANOVA: Analysis of variance
APN: Adiponectin
APP: Amyloid precursor protein
Aβ: β-Amyloid
AβO: Aβ oligomer
BBB: Blood–brain barrier
CNS: Central nervous system
DAPI: 4′,6-Diamidino-2-phenylindole
DMEM/F-12: Dulbecco’s modified Eagle’s medium
DMSO: Dimethyl sulfoxide
eIF2α: Eukaryotic translation initiation factor 2α
ELISA: Enzyme-linked immunosorbent assay
FBS: Fetal bovine serum
FoxO: Forkhead Box O
gAPN: Globular adiponectin
GFAP: Glial fibrillary acidic protein
HMW: High molecular weight
Iba1: Ionized calcium binding adaptor molecule-1
IL-1β: Interleukin-1β
LTP: Long-term potentiation
NF-kB: Nuclear factor kappa B
p-AMPK: Phosphorylated-5′AMP-activated protein kinase
PBS: Phosphate-buffered saline
PGC-1훼: Peroxisome proliferator-activated receptor 훾 co-activator 1-훼
PKR: Double-stranded RNA-dependent protein kinase
PPAR-γ: Peroxisome proliferator-activated receptor (PPAR)-γ
RT: Room temperature
SH-SY5Y: Human neuroblastoma cells
siRNA: Small interference RNA
SIRT1: Silent information regulator 1
TNFR1: Tumor necrosis factor type 1 death receptor
TNFα: Tumor necrosis factor α
TREM2: Triggering receptor expressed on myeloid cells 2
