# Supplementary figures and images for: Adiponectin suppresses amyloid-β oligomer (AβO)-induced inflammatory response of microglia via AdipoR1-AMPK-NF-κB signaling pathway
Source: J Neuroinflammation. 2019 May 25;16:110. doi: 10.1186/s12974-019-1492-6 (PMC6535190; doi:10.1186/s12974-019-1492-6)

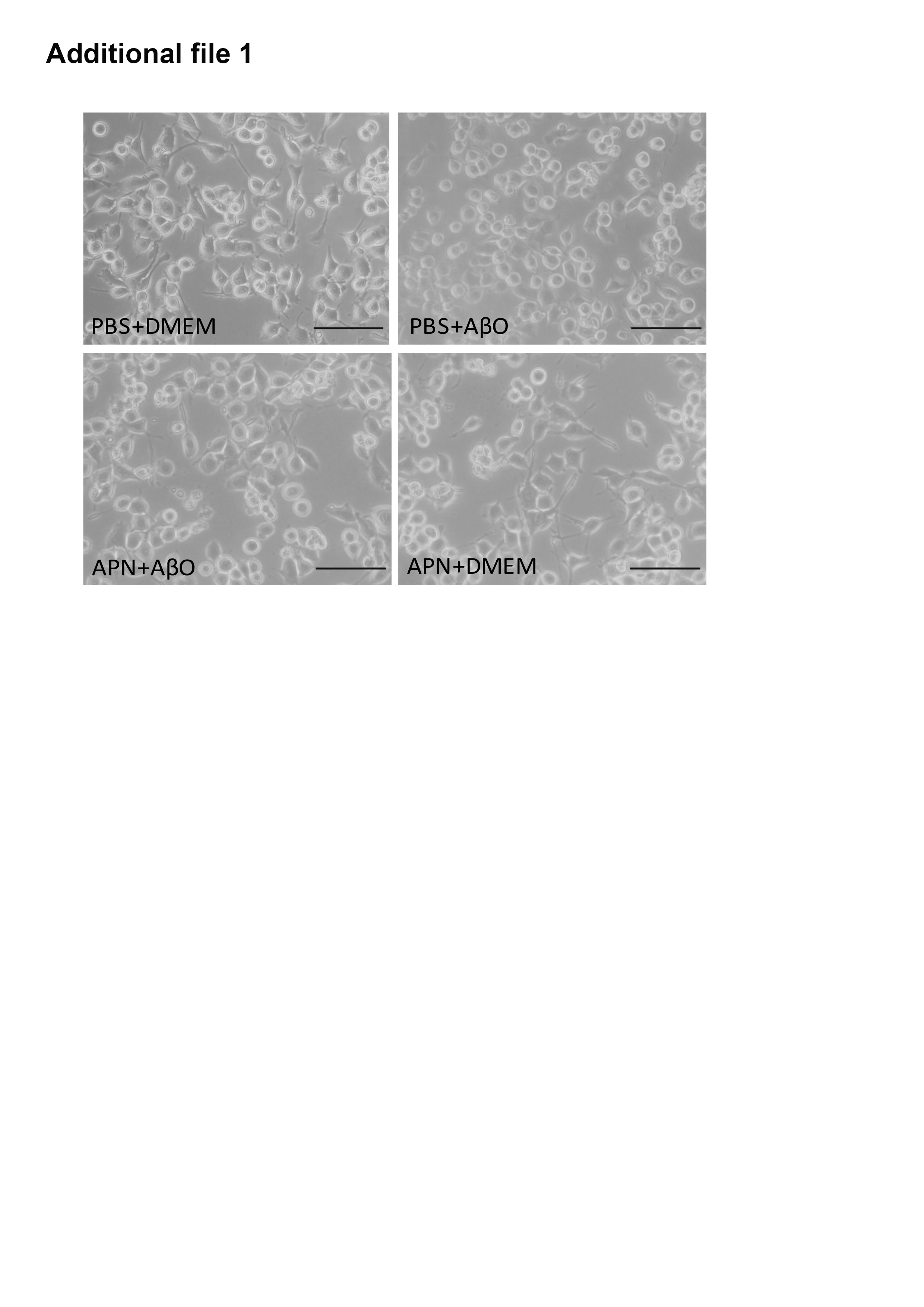

Supplement: Supplementary file 1 — APN treatment induced morphological changes of AβO-treated BV2 cells. Representative images were depicting morphology of BV2 cells pretreated with APN (10 μg/ml) for 2 h prior to incubation with AβO (10 μM) for an additional 24 h. Photomicrographs were taken directly from culture plates by phase-contrast microscopy. At the quiescent state, the BV2 microglia showed the typical ramified shape. Incubation with AβO of BV2 cells revealed an amoeboid shape which cell body was enlarged and extended processes were lost. Pretreatment with APN converted the amoeboid morphology of AβO-stimulated BV2 cells to a ramified morphology. Three independent experiments were performed. Scale bar 200 μm. (TIF 1630 kb) [file 12974_2019_1492_MOESM1_ESM.tif]

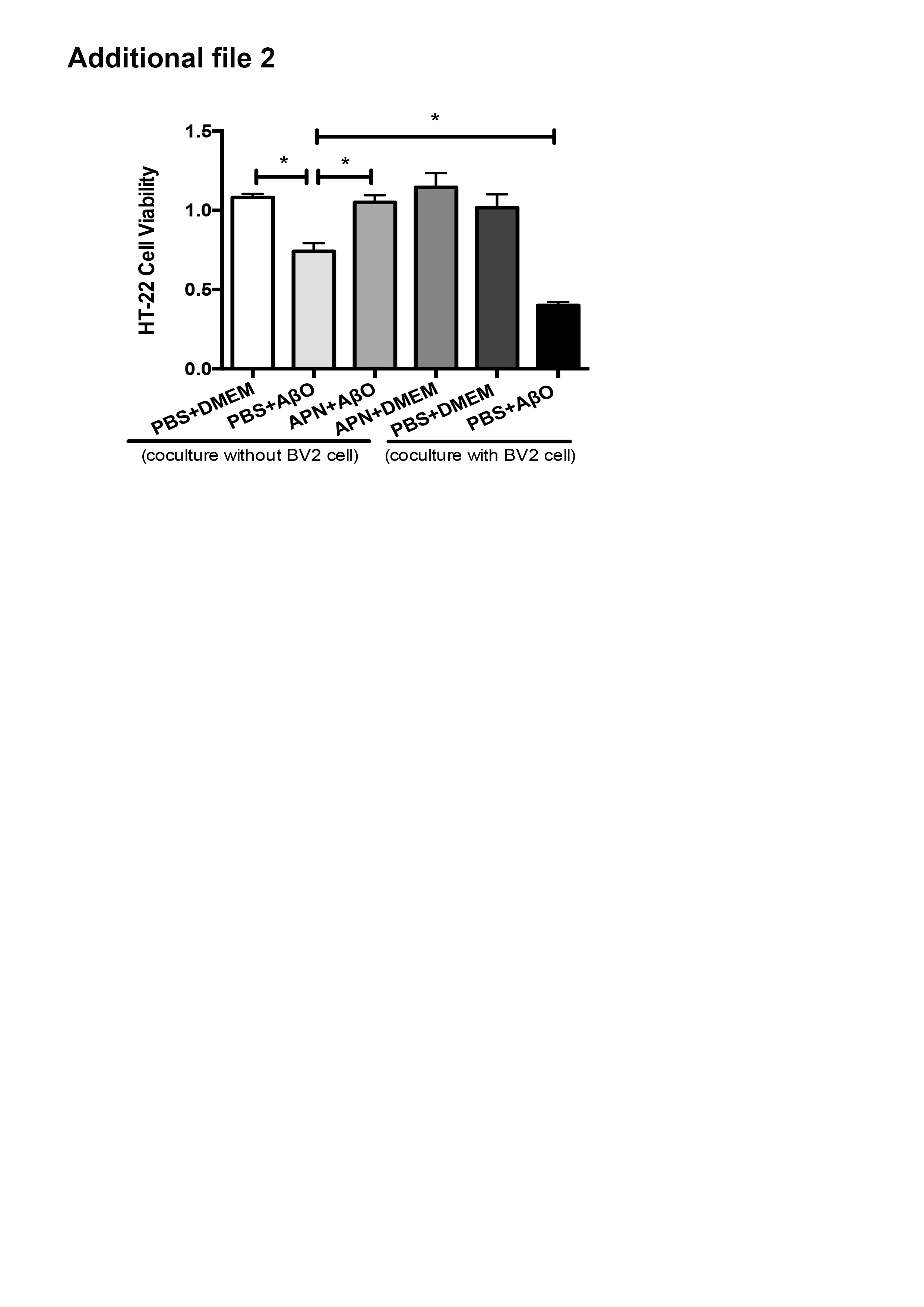

Supplement: Supplementary file 2 — BV2 microglia in co-culture system exacerbated neuronal loss under AβO exposure. HT-22 cells were treated with AβO with or without pre-treatment of APN, compared with HT-22 cells co-cultured with AβO-exposed BV2 cells in a transwell system. Data were presented as the mean ± SEM for at least three independent experiments, and each performed in triplicates (n = 3). One-way ANOVA with Tukey’s multiple comparison test revealed a difference between groups. *p < 0.05. (TIF 199 kb) [file 12974_2019_1492_MOESM2_ESM.tif]

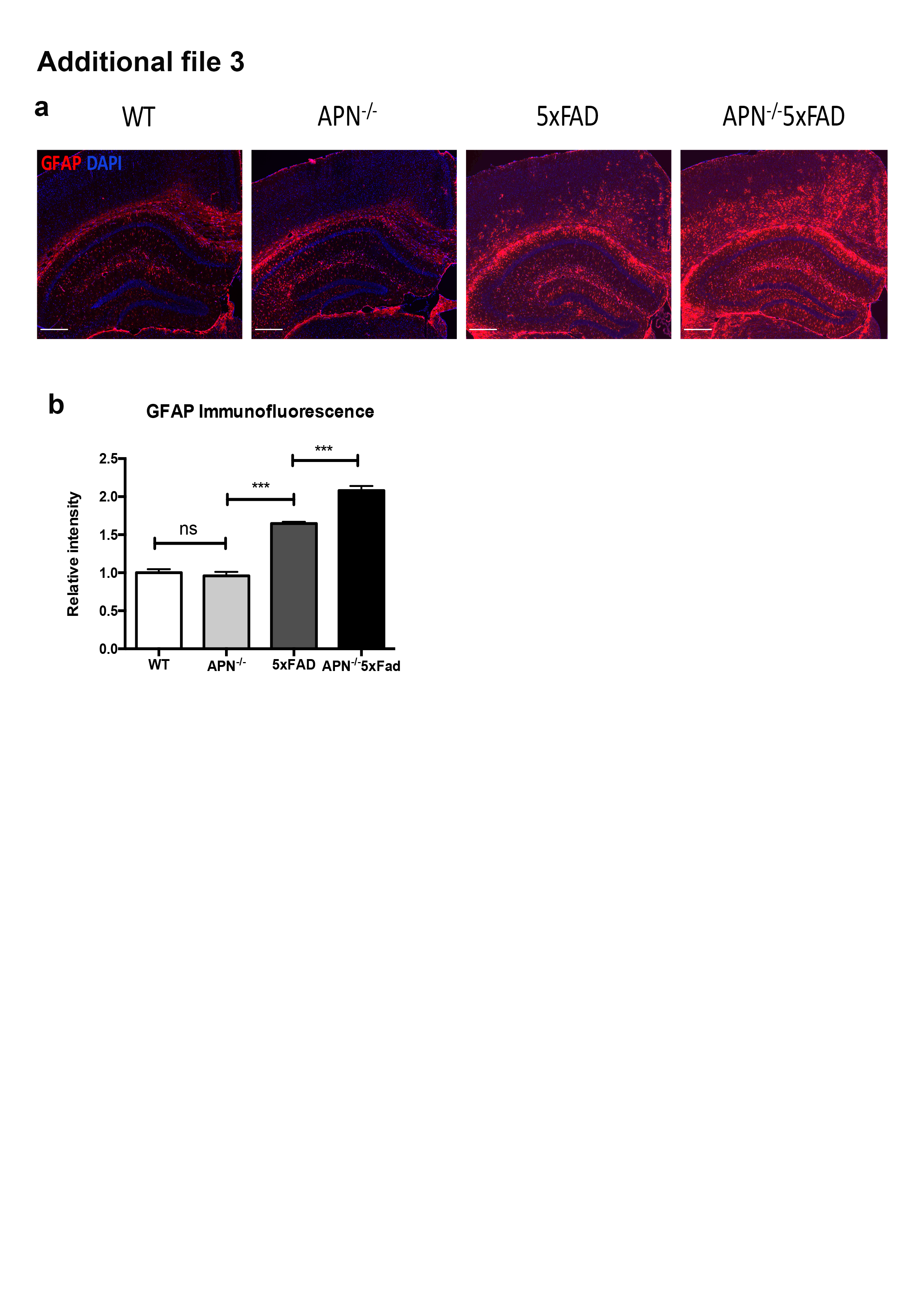

Supplement: Supplementary file 3 — APN deficiency exacerbated astrogliosis in 5xFAD mice. (a) Representative images of GFAP immunoreactivity of astrocytes in the cortex and hippocampus of WT mice, APN−/−mice, 5xFAD mice, and APN−/−5xFAD mice at 9 months old. Scale bar 400 μm. (b) Quantification of GFAP fluorescence intensity WT mice, APN−/−mice, 5xFAD mice, and APN−/−5xFAD mice (n = 4). One-way ANOVA with Tukey’s multiple comparison test revealed the difference between groups. **p < 0.01, ***p < 0.001; ns, statistically not significant. (TIF 3235 kb) [file 12974_2019_1492_MOESM3_ESM.tif]

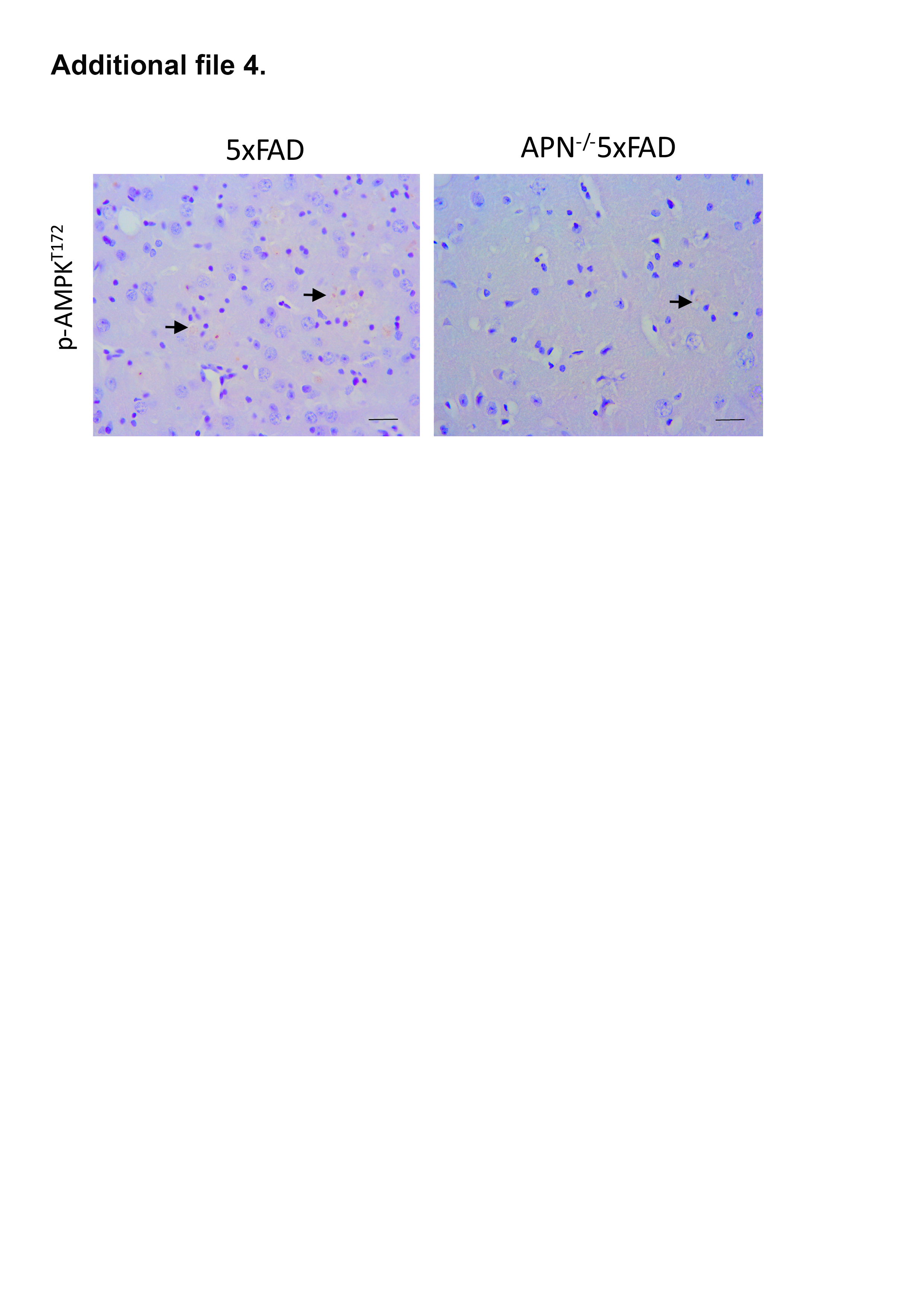

Supplement: Supplementary file 4 — APN deficiency reduced the level of phosphorylated AMPK in 5xFAD mice. (a) Representative image of immunohistochemistry staining of p-AMPKT172 (black arrows) in the cortex of 5xFAD mice and APN−/−5xFAD mice at 9 months old. Scale bar 200 μm. (TIF 2716 kb) [file 12974_2019_1492_MOESM4_ESM.tif]

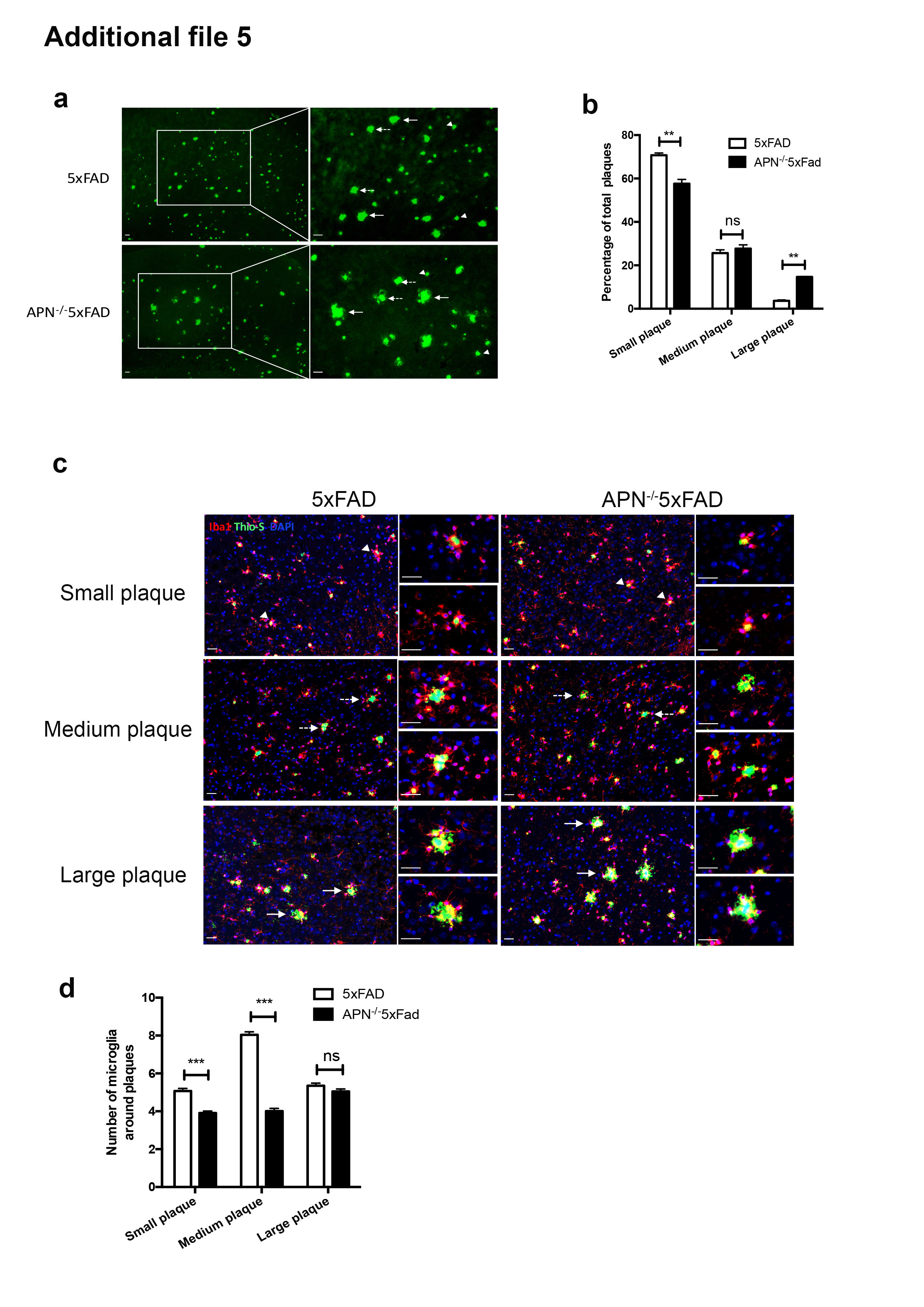

Supplement: Supplementary file 5 — APN deficiency increased the amyloid plaque size and decreased microglia clustering around amyloid deposits in 5xFAD mice. (a) Representative images of thioflavin-S-labeled amyloid plaque (green) in the 5xFAD mice and APN−/−5xFAD mice. Scale bar 30 μm. (b) Quantification of the percentage of thioflavin-S-labeled amyloid plaques according to their plaque size (small plaque radius < 10 μm, arrowheads; 10 μm < medium plaque radius < 15 μm, dashed arrows; large plaque radius > 15 μm, solid arrows) in the cortex (10 sections/mouse; n = 2). (c) Representative images of Iba1-labeled microglia (red) surrounding different size of thioflavin-S-labeled amyloid plaque (green) in the 5xFAD mice and APN−/−5xFAD mice. Images on the right represented magnified portion of microglia clustering around amyloid plaque (× 400). Scale bar 30 μm. (d) Quantification of the number of Iba1-labeled microglia within a 25-μm radius from different size of plaque edge (n = 80 plaques from 2 mice per genotype). Data were presented as the mean ± SEM. Two-way ANOVA with Tukey’s multiple comparison test revealed a difference between groups. **p < 0.01, ***p < 0.001; ns, statistically not significant. (JPG 768 kb) [file 12974_2019_1492_MOESM5_ESM.jpg]
